# Curcumin prevents dexamethasone-induced activation of the pseudorabies virus in rat pheochromocytoma cells through the miR-155-5p-Aak1-Numb/Notch2 signalling axis

**DOI:** 10.1186/s13567-025-01509-9

**Published:** 2025-04-21

**Authors:** Naixiu Wang, Fan Yang, Zhiyun Qiu, Lin Zhang, Dingqiu Zou, Yanru Tang, Ruihan Zhang, Chenlu Sun, Pei Liu, Kexin Qi, Jingyi Wang, Hua He, Ling Gan

**Affiliations:** 1 College of Veterinary Medicine, Southwest University, Chongqing, 402460 China; 2 College of Animal Science and Technology, Sichuan Agricultural University, Sichuan, 611130 China

**Keywords:** DEX, miR-155-5p/Aak1, pseudorabies virus, curcumin

## Abstract

Pseudorabies virus (PRV) causes neurological disorders and organ damage in diseased animals. After initial infection, PRV activity is gradually inhibited; however, stress stimulation increases the host’s glucocorticoid levels, which overcomes the inhibition of PRV activity. Curcumin (Cur) helps maintain the inhibitory state of the Epstein-Barr virus, although further research is needed to establish whether Cur can prevent PRV activation triggered by stress hormones. In this study, we used PC-12 cells to determine the effects of Cur on PRV activation. The cells were successfully infected with PRV at a multiplicity of infection of 1 for 24 h, resulting in the inhibition of PRV activity. Following incubation with 0.5 µM dexamethasone (DEX) for 4 h, the inhibition of PRV activity was blocked. Further mechanistic analyses using a dual-luciferase assay revealed that miR-155-5p directly targets and regulates Aak1 and its downstream signalling molecules, Numb and Notch2, in maintaining and disrupting PRV inhibition. Moreover, in vitro experiments using miR-155-5p mimics and inhibitors, combined with Aak1 overexpression and interference, confirmed that the miR-155-5p-Aak1-Numb/Notch2 axis prevented DEX-induced disruption of PRV inhibition by Cur. These findings provide a novel regulatory target for preventing stress-activated PRV and provide evidence for the potential use of Cur as a stress modulator in practical applications.

**Supplementary Information:**

The online version contains supplementary material available at 10.1186/s13567-025-01509-9.

## Introduction

Pseudorabies (PR), primarily characterised by encephalomyelitis, is an infectious disease caused by the pseudorabies virus (PRV) [1]. Although PRV activity becomes gradually suppressed, it remains latent in the neurons of hosts that tolerate primary infection. Furthermore, the pathogenicity and virulence of PRV increase when the host is exposed to stressors such as transportation, noise, and high temperatures [2]. It is widely recognised that domestic pigs are often affected by stressors, including environmental changes, transportation, weaning, and vaccination. Therefore, preventing stress-induced activation of PRV is crucial. A stress-induced transcription factor, Slug, enhances viral replication in neuro-2a cells infected with low doses of herpesvirus-1 (HSV-1) (a multiplicity of infection (MOI) = 1) [3]. In PC-12 cells infected with PRV (MOI = 1), PRV activity is inhibited, but treatment with 0.5 µM dexamethasone (DEX) blocks PRV suppression [4]. Studies have also shown that differentiated PC-12 cells derived from a rat neuronal cell line are comparable to neurons [5, 6]. Furthermore, glucocorticoids, steroid hormones produced by the adrenal cortex or inter-renal tissue cells, are also key hormones in stress response [7]. For example, the synthetic glucocorticoid DEX is frequently used as an activator in studies on herpes virus activation [8–10].

MicroRNAs (miRNAs) target and regulate mRNA levels during interactions between viral pathogens and host cells [11]. Numerous studies have also indicated that miRNAs play a role in repressing and activating herpes viruses by targeting and regulating their mRNAs. For instance, miR-101 can target host cells’ endogenous ATPase β subunit (ATP5B) to inhibit HSV-1 replication [12–14]. Notably, the endocytic-related genes Aak1, Numb, and Notch2 are key regulators of viral infection. Aak1, particularly, is involved in various physiological and pathological processes by regulating Numb and Notch2 levels [15–21]. Furthermore, Aak1 is an AP2-related protein kinase 1 named for its ability to phosphorylate the 156^th^ sub-unit of AP2 at Thr2 and participate in clathrin-mediated endocytosis (CME) [22–25].

Curcumin (Cur), an acidic phenolic compound derived from turmeric, has multiple pharmacological activities [26–29] and demonstrates high bioavailability after encapsulation [30–32]. Once absorbed from the gastrointestinal tract, Cur is mainly metabolised through reduction and binding to glucuronic acid and sulfuric acid, allowing for rapid distribution in the body [33–35]. Due to its high lipid solubility and permeability across the blood–brain barrier, Cur is widely used in preventing and treating nervous system diseases [36]. Previous studies have shown that Cur inhibits HSV and Epstein-Barr virus (EBV) [37] reactivation while effectively stabilising mitochondrial membrane potential (MMP) and protecting neurons [38].

Additionally, miR-155-5p has been identified as a downstream target of Cur in regulating the proliferation and apoptosis of salivary gland tumour cells [39]. However, it remains unclear whether Cur prevents glucocorticoid-induced disruption of PRV by regulating the miR-155-5p-Aak1 pathway. In this study, we investigated the relationship between miR-155-5p and Aak1 and the contribution of miR-155-5p-Aak1-Numb/Notch2 in regulating glucocorticoid-activated PRV by Cur.

## Materials and methods

### Cell culture

Highly differentiated PC-12 and PK-15 cell lines (purchased from the Cell Bank of the Chinese Academy of Sciences) were cultured in Dulbecco’s modified Eagle’s medium (DMEM; C11995500BT; Gibco, USA) and supplemented with 10% foetal bovine serum (FBS; 10099141C; Gibco, USA) and 1% penicillin/streptomycin (15140122; Gibco, USA).

### Experimental design

After incubation and passaging in a humidified incubator at 37 °C with 5% CO_2_, PC-12 cells were inoculated into 35 mm glass-bottomed confocal petri dishes and 6-, 24-, and 96-well plates. When the cells in the dishes or plates grew to approximately 80% confluence, 750, 500, 150, and 100 μL of PRV viral solution (MOI = 1) was inoculated into each well. The blank control group (CON) and 0.5 µM DEX-treated group (DEX) were treated with a 2% cell maintenance solution for the control culture. The cells in the PRV + DEX group were incubated with PRV at MOI = 1 for 24 h and then treated with 0.5 µM DEX culture solution for 4 h. Simultaneously, the DEX group was treated with 0.5 µM DEX culture solution for 4 h. The CON and PRV groups were treated with the same volume of serum-free DMEM high-glucose medium and cultured for 4 h. Cur (A600346, Sangon Biotech, China), miRNA mimics and inhibitors (Gemma, China), Aak1-OE (Yunzhou Biotech, China), and shRNAs (Shanghai Gemma, China) were used for combination treatment in serum-free medium. For plaque assay, PC-12 cell culture supernatants were collected to measure the viral copy number and viral particle content in PK-15 cells. Cell viability was assessed using the CCK-8 assay (BS12B, Biosharp, China). Reactive oxygen species (ROS) levels were measured by a microplate assay (E004-1-1, Nanjing Jianjian, China), and mitochondrial membrane potential (MMP) was evaluated using a fluorescence assay (JC-1, BL726A, Biosharp, China). Lastly, a cellular immunofluorescence assay was conducted to detect the amount of PRV protein in cells. All procedures were performed per the manufacturer’s protocols.

### Transfection of miR-155-5p mimic and inhibitor, Aak1-OE, and shRNA

The miR-155-5p mimic (sense: UUAAUGCUAAUUGUGAUAGGGGU; antisense: CCCUAUCACAAUUAGCAUUUAAUU) and repressor (sense: ACCCCUAUCACAAUUAGCAUUUAA; Jimma Pharmaceuticals, China) were designed using overexpression vectors (Yun Zhou Biotechnology, China). Short hairpin RNA (shRNA) fragments of Aak1 that effectively inhibited its translation (sense: 5′-CACCGATGGGAAGTTCTCATTCTCATTTCAAGAGAATGAGAAT GAGAACTTCCCATTTTTTTTTG-3′ and antisense: 5′-GATCCAAAAAAAAATGGGAAGTTC TCATTCTCATTCTCTCTTGAAATGAGAATGAGAACTTCCCATC-3′; Jimma Pharmaceuticals, China) were also designed. Western blotting was employed to evaluate the inhibitory efficiency of the shRNA probe at the Aak1 protein level. PC-12 cells were cultured for 18–24 h until 60–80% cell confluence. Transfection was performed using GP-transfect-Mate (G04008; Gemma Pharmaceuticals, China). The final concentrations of miR-155-5p mimic and inhibitor were 40 nM and 160 nM, respectively. Aak1-OE and shRNA concentrations were 1 μg/mL, and the finalised transfection reagent (GP-transfect-Mate) concentration was 2 μL/mL.

### Western blotting

PC-12 cell samples were lysed in an ice-cold radioimmunoprecipitation assay buffer (RIPA; BL504A, Biosharp), 1 × complete protease inhibitor (BL612A; Biosharp), and a phosphatase inhibitor cocktail (BL615A; Biosharp). Cell homogenates (20 μg/well) were loaded onto 8, 10, or 12% sodium dodecyl sulfate (SDS) polyacrylamide gels under denaturing conditions. Proteins were resolved electrophoretically at 100 mA for 90 min and transferred onto a 0.45 µM polyvinylidene fluoride (PVDF) membrane (Millipore, USA) at 200 V for 60 min (Power Pack; Bio-Rad Laboratories, USA). Next, the membrane was blocked with 5% non-fat dry milk or 5% bovine serum albumin (BSA) in tris-buffered saline containing Tween-20 (TBST) at 25 ℃ and then incubated overnight at 4 °C with the following primary antibodies: GAPDH (1:5000; Proteintech, China), PRV (1:500; Thermo Fisher Scientific, USA), Aak1 (1:2000; Huaan Biotech, China), Numb (1:2000; Huaan Biotech, China), and Notch2 (1:1000; Wanleibio, China). Secondary antibodies (HRP-conjugated Goat Anti-Rabbit IgG (H + L) and HRP-conjugated Goat Anti-Mouse IgG(H + L); 1:5000; Proteintech) were then added. Incubation was at 25 ℃ for 2 h. An enhanced chemiluminescence kit (P0018AS, Beyotime, China) was used to visualise the immunoblots. Immunoreactive bands were analysed using Image J software (NIH, Bethesda, MD, USA).

### Total RNA extraction and real-time quantitative polymerase chain reaction

RNA was extracted from PC-12 cells using Trizol reagent (B511311, Sangon Biotech, China) according to the manufacturer’s instructions, with three biological replicates per group. PerfectStart^®^ The Uni RT & qPCR Kit (AUQ-01, TransGen Biotech, China) was used to synthesise cDNA using 1000 ng of total RNA. cDNA, primers. PerfectStart Green qPCR SuperMix and nuclease-free water were combined into a 20 µL reaction system to perform quantitative real-time polymerase chain reaction (PCR) in a real-time PCR system (QuantStudio 5, Thermo Scientific, USA). All operational steps were completed under the manufacturer’s guidance, and GAPDH was used as an internal reference. The primer sequences are listed in Table 1.

**Table 1:** **Fluorescent primer sequences**

Gene name	Primer sequence	Size (bp)
gB	F:5′- GTCACCCGCGTGCTGATCGTCT -3′ R:5′- GGCAACCACCGGCGCTACTTT -3′	107
gE	F:5′- CTCTGCGTGCTGTGCTCCC-3′ R:5′-CCTCCTCGTCGTCGTCGTC -3′	145
IE180	F:5′- TCGTGCGCCTCATCTACAG -3′ R: 5′- TGGCAGAACTGGTTGAAGCG -3′	100
GAPDH	F:5′-GACATGCCGCCTGGAGAAAC-3′ R:5′-AGCCCAGGATGCCCTTTAGT-3′	92
Wee1	F:5′-TTGCTCTTGCTCTCACAGTCGTATG-3′ R:5′-AGCACTTGTGGAATCCGAGGTAATC-3′	112
CEBPB	F:5′-GCTGAGCGACGAGTACAAGATGC-3′ R:5′-CTTGTGCTGCGTCTCCAGGTTG-3′	100
Aak1	F:5′-ATTCAGCCAGCCCTGACTCC-3′ R:5′-AGGCTAGGCTGATTGCTGGG-3′	83

### Plaque assay

PK-15 cells were resuspended from well-grown monolayer culture bottles, adjusted to the appropriate concentration, and transferred to 12-well plates at a density of 1 mL of cell suspension per well. The cells were then cultured until the cell monolayer was fully established. The previous period’s cell culture supernatants from the different treatment groups were dissolved in ice. The PK-15 cell culture medium was discarded, and cells were washed with 1 × PBS buffer. The cell culture supernatants were added to each well and incubated at 37 ℃ and 5% CO_2_ for 2 h. Afterwards, 0.6% autoclaved low melting point agarose was held in a water bath at 55 °C, mixed in equal volumes with 2% cell maintenance solution preheated at 37 °C, and formulated as nutrient layer agarose. Once the temperature of the nutrient agarose layer fell below 42 ℃, it was quickly added to the culture wells and placed at 25 °C until solidification. Then, the cell culture dish was gently inverted and put into a 37 ℃, 5% CO_2_ incubator for culture. When lesions appeared, 1 mL of 1% formaldehyde solution was added to each well. Overnight incubation was maintained at 37 ℃. Subsequently, the agar layer was removed, and 0.15% crystal violet was added for 5 min to visualise the empty spots.

### Extraction of viral DNA

Cell culture supernatants from different treatment groups were extracted into new centrifuge tubes following the instructions provided for the viral DNA extraction reagent (B518270, Sangon Biotech, China) for DNA extraction. The quality of the extracted DNA was assessed using an ultra-micro spectrophotometer (NanoPhotometer, Implen, Germany). Once the DNA met the quality standards, it was stored at −20 °C for future use.

### Detection of PRV protein content in cells using cellular immunofluorescence assay

Mature, well-growing PC-12 cells were transferred into a 35 mm glass substrate confocal culture dish (D35-14-1.5P, Cellvis, USA) and incubated until the cells reached approximately 80% confluence. After viral infection and DEX treatment, the cells were removed from the incubator and washed thrice with 1 × PBS buffer. Then, 1 mL of pre-cooled 4% paraformaldehyde at 4 °C was added to each cell culture well and fixed at 25 ℃ for 15 min. After fixation, the fixing solution was removed, and cells were washed thrice using 1 × PBS buffer for 5 min each. We then added 1 mL of 0.5% Triton X-100 to each well, which was allowed to penetrate for 20 min at 25 ℃. The Triton X-100 was removed by washing cells thrice with 1 × PBS buffer for 5 min each. BSA at 1 mL of 5% was added to each well and sealed at 25 ℃ for 30 min. The sealing liquid was removed, and the wells were washed thrice with 1 × PBS buffer for 5 min each.

Next, 0.5 mL of PRV primary antibody (diluted with 1% BSA 100:1 antibody) was added to each well and placed in a wet box at 4 ℃ overnight. The cells were washed thrice with 1 × PBS buffer for 5 min each to remove the unbound PRV primary antibody. A secondary antibody of 0.5 mL was added to each well (1:500 dilution) and incubated at 25 ℃ in the dark for 2 h. Next, the cells were washed thrice with 1 × PBS buffer for 5 min each to remove excess secondary antibodies. Finally, 0.5 mL of anti-DAPI was added to each well, and staining was performed at 25 ℃ for 3–5 min. The DAPI staining solution was discarded, and the cells were immediately washed thrice with 1 × PBS buffer for 5 min each. Images were captured using a fluorescence-inverted microscope.

### Statistical analysis

All statistical analyses were performed using GraphPad Prism 7.0 and SPSS 26.0 software. The mean ± standard deviation (SD) of all biological replicates was used to create graphs. One-way analysis of variance (ANOVA) was employed to compare differences between groups, and an independent sample *t*-test was used to compare differences between two groups. The effect size was evaluated using η^2^. Differences between groups were considered significant at *P*-values < 0.05 (*), < 0.01 (**), < 0.001 (***), and < 0.0001 (****).

## Results

### DEX activates PRV in PC-12 cells

**Figure 1:**
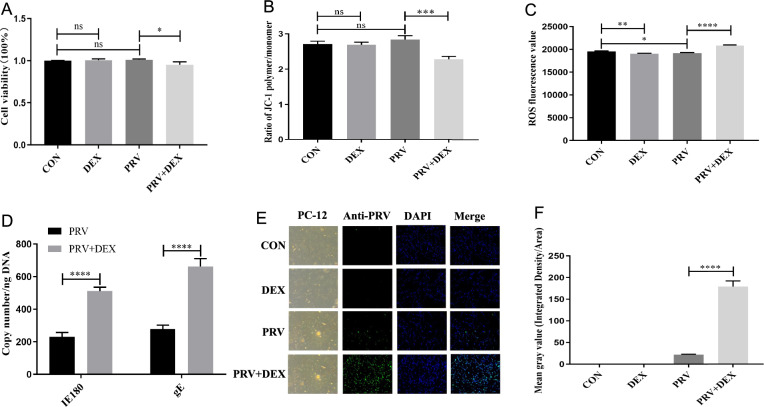
**Effects of DEX treatments on PC-12 cell viability, mitochondrial function, and virus replication-related indicators.** **A** Cell viability (η^2^ = 0.694); **B** Mitochondrial membrane potential (MMP) (η^2^ = 0.895); **C** ROS content (η^2^ = 0.983); **D** Virus copy number (η^2^ = 0.979, η^2^ = 0.974); **E** PRV protein level determined by IFA (10 × 10); **F** PRV protein quantification map (greyscale value) (η^2^ = 0.995). Mean ± SD, *n* = 3, *P < 0.05, **P < 0.01, ***P < 0.001, ****P < 0.0001, ns: not significant. CON: blank control; DEX: 4 h incubation with 0.5 µM DEX; PRV: PRV infection with MOI = 1 for 24 h; PRV + DEX: PRV with MOI = 1 was infected for 24 h and then incubated with 0.5 µM DEX for 4 h.

PC-12 cells were infected with PRV at MOI = 1 for 24 h, followed by incubation with 0.5 µM DEX for 4 h. Compared to the CON group, there were no significant changes in cell viability (*P* = 0.464) (Figure 1A) and MMP levels (*P* = 0.116) (Figure 1B). However, the ROS content (*P* = 0.003) (Figure 1C) significantly decreased, while the virion levels (Additional file 1) in the PC-12 cells of the PRV group did not change substantially. In contrast, compared with the PRV group, the PRV + DEX group presented a significant decrease in cell viability (*P* = 0.0052) (Figure 1A) and MMP level (*P* < 0.0001) (Figure 1B), along with increased ROS content (*P* < 0.0001) (Figure 1C). In addition, the copy numbers of the viral genes I*E180* and *gE* (*P* < 0.0001, *P* < 0.0001) (Figure 1D), virus release (Additional file 1) and virus protein level (*P* < 0.0001) (Figures 1E and F) also significantly increased in the PRV + DEX group. These findings indicate that the PRV activity of PC-12 cells infected with MOI = 1 PRV for 24 h was inhibited, and this inhibitory state was disrupted when PRV-infected PC-12 cells were incubated with 0.5 µM DEX.

### miR-155-5p participates in the maintenance and destruction of inhibited PRV through targeted regulation of the Aak1 gene

**Figure 2:**
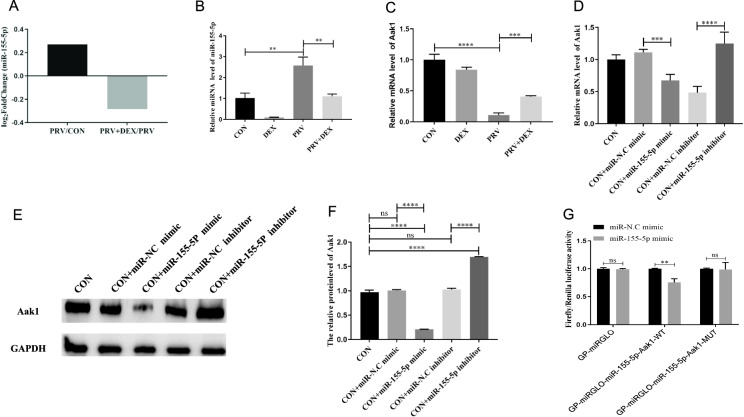
**Targeted regulation of Aak1 gene by miR-155-5p during the maintenance and destruction of relative inhibited PRV activity**. **A** miRNA-Seq results; **B** RT-qPCR validation of miRNA expression results (η^2^ = 0.983); **C** RT-qPCR validation of Aak1 mRNA level (η^2^ = 0.983); **D** The mRNA level of *Aak1* gene after transfection with miR-155-5p mimic/inhibitor (η^2^ = 0.897); **E** Using GAPDH as the internal reference Aak1 protein level was detected by western blotting after transfection with miR-155-5p mimic/inhibitor; **F** Relative level of Aak1 protein (η^2^ = 0.998); **G** Dual-luciferase reporter gene results (η^2^ = 0.019, η^2^ = 0.911, η^2^ = 0.08). Mean ± SD, *n* = 3, *P < 0.05, **P < 0.01, ***P < 0.001, ****P < 0.0001, ns: not significant.

RNA-Seq screening of differentially expressed miRNAs (accession number: GSE202138) revealed that compared to the CON group, the levels of miR-155-5P in PC-12 cells were up-regulated in the PRV group but down-regulated in the PRV + DEX group (Figure 2A). These results suggest that miR-155-5p is involved in the maintenance and disruption of the PRV-inhibition state. This finding was further validated by RT-qPCR (Figure 2B). Subsequently, using Targeted Scan and miRDB software, the target genes of miR-155-5p were predicted to be *Wee1*, *CEBPB*, and *AaK1* [40, 41]. The results of the RT-qPCR indicated that the miR-155-5p level was negatively correlated with the levels of the three target genes (Figure 2C and Additional file 2). To further confirm the regulatory role of miR-155-5p on these three target genes in maintaining and disrupting the PRV-inhibition state, we transfected miR-155-5p mimics and inhibitors into PC-12 cells.

RT-qPCR was subsequently employed to detect the miR-155-5p (Additional file 3A) and the mRNA levels of the three target genes (Figure 2D and Additional files 3B, C). The results showed that only Aak1 levels had a negative regulatory relationship with miR-155-5p levels following the transfection of miR-155-5p mimics and inhibitors in PC-12 cells (Figure 2D). Afterwards, we transfected miR-155-5p mimics and inhibitors to verify the expression profile of Aak1 at the protein level. Our findings showed that the levels of Aak1 protein were negatively correlated with the levels of miR-155-5p (Figures 2E and F). The dual-luciferase reporter assay confirmed the direct regulatory relationship between miR-155-5p and Aak1.

Lastly, a dual-luciferase reporter gene vector was constructed (Additional file 4), which demonstrated that overexpression of miR-155-5p significantly increased the fluorescence activity of the GP-miRGLO-miR-155-5p-Aak1-WT vector (*P* = 0.003) but did not influence the fluorescence activity of the GP-miRGLO-miR-155-5p-Aak1-MUT vector (*P* = 0.866) (Figure 2G).

These results indicate that miR-155-5p directly regulates the expression of the *Aak1* gene.

### The mediating role of miR-155-5p-Aak1 in the maintenance and destruction of inhibited PRV

To further investigate whether miR-155-5p-Aak1 is involved in the maintenance and destruction of inhibited PRV, we constructed an overexpression vector (Aak1-OE) and a shRNA interference plasmid for Aak1. Cellular immunofluorescence results showed successful transfection of the Aak1 overexpression plasmid (Additional file 5A). Compared to the blank control and the control plasmid, RT-qPCR (Additional file 5B) and western blotting results (Additional file 5C) indicated that the expression level of the *Aak1* gene was up-regulated in the Aak1-OE group at both the mRNA (*P* < 0.0001, *P* < 0.0001) and protein levels (*P* < 0.0001, *P* < 0.0001). This finding suggests that the Aak1 overexpression plasmid exhibits a superior transfection effect and could be used for subsequent experiments. Cellular immunofluorescence analysis (Additional file 6A) confirmed the successful transfection of the Aak1 interference plasmid. The RT-qPCR results showed that the positive control had a significantly greater inhibitory effect (*P* = 0.0001) (Additional file 6B). Among the four interfering RNAs screened using RT-qPCR (Additional file 6C) and western blotting analysis (Additional file 6D), shRNA2 (*P* < 0.01, *P* < 0.001) and shRNA4 (*P* < 0.0001, *P* < 0.0001) showed better inhibitory effects on Aak1 expression compared to shRNA1 and shRNA3 at both the mRNA and protein levels. Furthermore, shRNA4 exhibited a stronger inhibitory effect compared to shRNA2. As a result, shRNA4 was selected for the subsequent experiments.

**Figure 3:**
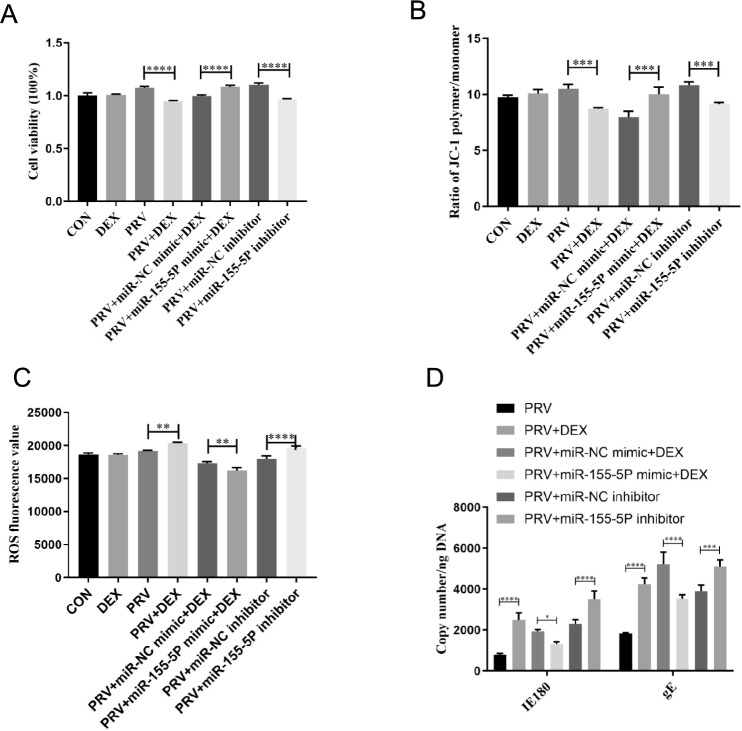
**Effects of miR-155-5P on the function of PC-12 cells**. **A** Cell viability (η^2^ = 0.956); **B** Mitochondrial membrane potential (MMP)(η^2^ = 0.894); **C** ROS content (η^2^ = 0.967); **D** Virus copy number (η^2^ = 0.951); Mean ± SD, *n* = 3, *P < 0.05, **P < 0.01, ***P < 0.001, ****P < 0.0001, ns: not significant.

We then transfected the miR-155-5p inhibitor, Aak1-OE, and the corresponding control in the cells of the PRV group and the miR-155-5p mimic, shRNA, and the corresponding control in the PRV + DEX group. In the PRV group, the transfection of miR-155-5p inhibitor in PC-12 cells resulted in decreased cell viability (*P* < 0.0001) (Figure 3A) and cellular MMP (*P* < 0.001) (Figure 3B) compared to the corresponding negative controls. Additionally, this transfection resulted in an increase in cellular ROS (*P* < 0.0001) (Figure 3C) and the copy number of the viral genes IE180 (*P* < 0.0001) and gE (*P* < 0.001) (Figure 3D). Meanwhile, in the PRV + DEX group, transfection with miR-155-5p mimics resulted in increased cell viability (*P* < 0.0001) (Figure 3A) and cellular MMP (*P* < 0.001) (Figure 3B). Additionally, this transfection led to a decrease in ROS levels (*P* < 0.002) (Figure 3C) and the replication of viral genes IE180 (*P* < 0.001) and gE (*P* < 0.0001) (Figure 3D).

**Figure 4:**
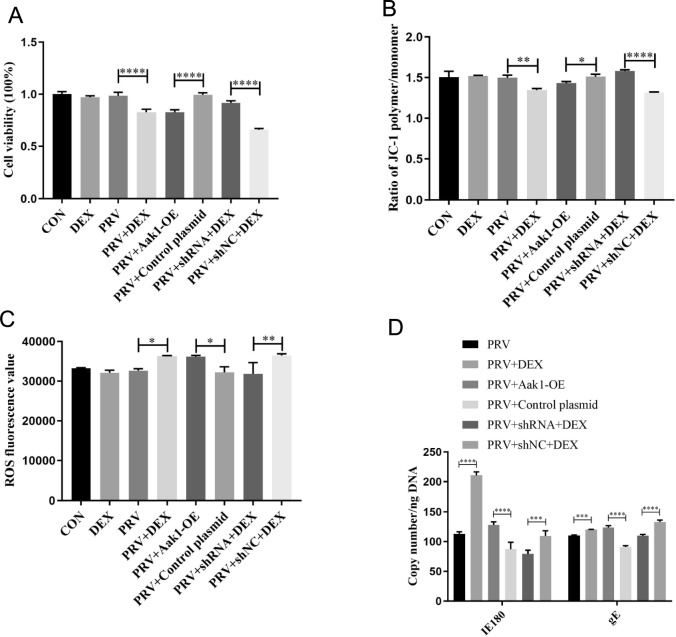
**Effects of Aak1 on the function of PC-12 cells.** **A** Cell viability (η^2^ = 0.973); **B** Mitochondrial membrane potential (MMP) (η^2^ = 0.912); **C** ROS content (η^2^ = 0.803); **D** Virus copy number (η^2^ = 0.981, η^2^ = 0.981); Mean ± SD, *n* = 3, *P < 0.05, **P < 0.01, ***P < 0.001, ****P < 0.0001, ns: not significant.

In PC-12 cells, transfection with the Aak1 overexpression plasmid in the PRV group decreased cell viability (*P* < 0.0001) (Figure 4A) and (*P* = 0.0198) and MMP level (Figure 4B) while increasing the ROS level (*P* = 0.0131) (Figure 4C) and the replication of the viral genes IE180 (*P* < 0.0001) and gE (*P* < 0.0001) (Figure 4D). In contrast, the PRV + DEX group showed that transfection with the Aak1 interference plasmid elevated cell viability (*P* < 0.0001) (Figure 4A) and MMP level (*P* < 0.0001) (Figure 4B) while decreasing the ROS level (*P* = 0.0036) (Figure 4C) and the replication of viral genes IE180 (*P* < 0.0001) and gE (*P* < 0.0001) (Figure 4D).

### The downstream regulatory molecules of miR-155-5p-Aak1 in the process of maintenance and destruction of inhibited PRV

**Figure 5:**
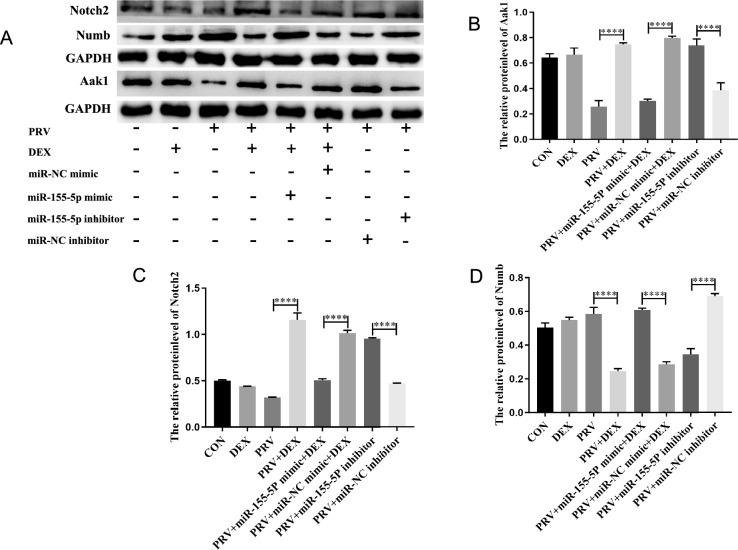
**Effects of miR-155-5p on Notch2 and Numb protein levels.** **A** The protein levels of Aak1, Numb and Notch2 in PC-12 cells transfected with miR-155-5p mimic and miR-155-5p inhibitor detected by western blotting, with GAPDH as an internal reference; **B** Relative protein level of Aak1 (η^2^ = 0.976); **C** Relative protein level of Notch2 (η^2^ = 0.993); **D** Relative protein level of Numb (η^2^ = 0.985). Mean ± SD, *n* = 3, *P < 0.05, **P < 0.01, ***P < 0.001, ****P < 0.0001, ns: not significant.

To further investigate the downstream regulatory molecules of miR-155-5p-Aak1 involved in the maintenance and destruction of inhibited PRV, we performed western blotting to detect Aak1, endocytosis-related proteins Numb, and Notch2 protein levels. The results indicated that following transfection of the miR-155-5p inhibitor in the cells of the PRV group, the protein levels of Aak1 (*P* < 0.0001) (Figures 5A and B) and Notch2 (*P* < 0.0001) (Figures 5A and C) were up-regulated. The protein level of Numb was down-regulated compared with the negative control (*P* < 0.0001) (Figures 5A and D). In contrast, after transfection of the miR-155-5p mimic in the PRV + DEX group, the protein levels of Aak1 (*P* < 0.0001) (Figures 5A and B) and Notch2 (*P* < 0.0001) (Figures 5A and C) protein levels were down-regulated. Numb protein levels were up-regulated compared with the negative control (*P* < 0.0001) (Figures 5A and D).

**Figure 6:**
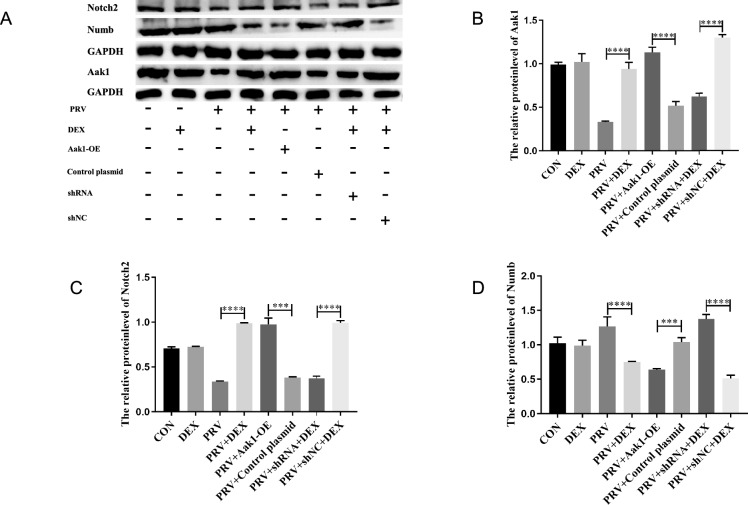
**Effects of Aak1 on Notch2 and Numb protein levels.** **A** The protein levels of Aak1, Numb, and Notch2 in PC-12 cells transfected with Aak1-OE and shRNA detected by western blotting, with GAPDH as an internal reference; **B** Relative protein level of Aak1 (η^2^ = 0.979); **C** Relative protein level of Notch2 (η^2^ = 0.993); **D** Relative protein level of Numb (η^2^ = 0.954). Mean ± SD, *n* = 3, *P < 0.05, **P < 0.01, ***P < 0.001, ****P < 0.0001, ns: not significant.

After transfection of Aak1 overexpression plasmid in the PRV group’s cells, the protein levels of Aak1 (*P* < 0.0001) (Figures 6A and B) and Notch2 (*P* < 0.001) (Figures 6A and C) were increased. In contrast, the levels of Numb decreased compared with the negative control group (*P* < 0.001) (Figures 6A and D). After transfection of Aak1 interference plasmid in the PRV + DEX group, the protein levels of Aak1 (*P* < 0.0001) (Figures 6A and B) and Notch2 (*P* < 0.0001) (Figures 6A and C) decreased, while the levels of Numb increased, compared with the negative control group (*P* < 0.0001) (Figures 6A and D). These results suggest that miR-155-5p-Aak1 mediates DEX-induced PRV activation by regulating Numb and Notch2 expression.

### Curcumin prevents DEX-induced PRV activation

**Figure 7:**
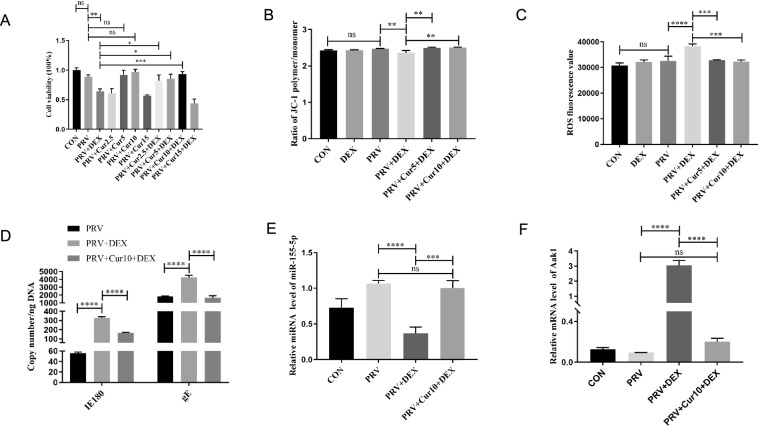
**Cur prevents DEX from activating PRV**. **A** Cell viability (η^2^ = 0.929); **B** Mitochondrial membrane potential (MMP) (η^2^ = 0.791); **C** ROS content; **D** Virus copy Number (η^2^ = 0.997, η^2^ = 0.975); **E** miR-155-5p level (η^2^ = 0.916); **F** Aak1 mRNA level (η^2^ = 0.989). Mean ± SD, *n* = 3, *P < 0.05, **P < 0.01, ***P < 0.001, ****P < 0.0001, ns: not significant. CON: blank control; DEX: 4 h incubation with 0.5 µM DEX; PRV: PRV infection with MOI = 1 for 24 h; PRV + DEX: PRV with MOI = 1 was infected for 24 h and then incubated with 0.5 µM DEX for 4 h. PRV + Cur5 + DEX and PRV + Cur10 + DEX: the cells were stimulated by DEX after incubation with 5 and 10 µM of Cur following PRV infection.

Considering previous studies, different concentrations of Cur were used to incubate PC-12 cells for 24 h to determine the toxicity range of Cur on PC-12 cells [42–44]. Cell viability analysis indicated that less than or equal to 30 µM Cur had no toxic effect on PC-12 cells (Additional file 7). The results indicated that incubation of cells in the PRV group with 5 µM and 10 µM Cur for 24 h did not affect cell viability compared to the PRV group (*P* = 0.565, *P* = 0.112). In contrast, cell viability in the PRV + DEX group was reduced (*P* = 0.0022). Compared to the PRV + DEX group, cell viability increased in the PRV + Cur5 + DEX (*P* = 0.0122) and PRV + Cur10 + DEX groups (*P* < 0.001) (Figure 7A). These findings suggest that 5 µM and 10 µM Cur prevented DEX from activating PRV.

Furthermore, compared to the PRV group, the MMP level in the PRV + DEX group decreased (*P* = 0.0062) (Figure 7B), while the ROS content (*P* < 0.0001) (Figure 7C) and the number of viral vacuoles increased (Additional file 8). In contrast, compared to the PRV + DEX group, the MMP levels decreased (*P* = 0.002, *P* = 0.001) (Figure 7B). ROS content was elevated (*P* < 0.001, *P* < 0.001) (Figure 7C), and there was no vacuole formation (Additional file 8) in the PRV + Cur5 + DEX and PRV + Cur10 + DEX groups. Moreover, 10 µM Cur was more effective in preventing DEX-induced PRV activation than 5 µM curcumin. Therefore, 10 µM Cur was used in subsequent experiments. The copy numbers of IE180 and gE were higher in the PRV + DEX group than those in the PRV group (*P* < 0.0001) (Figure 7D) and lower in the PRV + Cur5 + DEX group compared to the PRV + DEX group (*P* < 0.0001) (Figure 7D). These results suggest that 10 µM Cur prevents DEX-induced PRV activation.

The RT-qPCR analysis revealed that Cur exhibited an opposing trend in regulating the levels of miR-155-5p l (Figure 7E) and Aak1 mRNA l (Figure 7F). However, the differences between miR-155-5p (*P* = 0.493) and Aak1 mRNA levels (*P* = 0.448) were insignificant in the Cur incubation group compared to those in the PRV group. This outcome suggests that miR-155-5p-Aak1 mediates the prevention of DEX-activated PRV by Cur.

### MiR-155-5p-Aak1-Numb/Notch2 pathway mediates the role of Cur in preventing DEX-activated PRV

**Figure 8:**
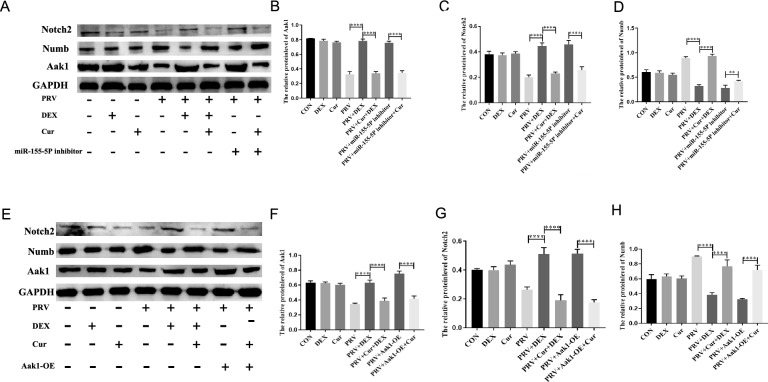
**The protein levels of Notch2, Numb, and Aak1 in the process of Cur preventing DEX-activated PRV**. **A** The protein levels of Aak1 (η^2^ = 990), Numb (η^2^ = 0.981), and Notch2 (η^2^ = 0.964) in PC-12 cells transfected with the miR-155-5p mimic/inhibitor were detected by western blotting, and the intensity was calculated using GAPDH as an internal control to normalise protein loading (**B**–**D**); **E** The protein levels of Aak1 (η^2^ = 969), Numb (η^2^ = 954), and Notch2 (η^2^ = 967) in PC-12 cells transfected with Aak1-OE were detected by western blotting, and intensity was calculated using GAPDH as an internal control to normalise protein loading (**F**–**H**). Mean ± SD, *n* = 3, *P < 0.05, **P < 0.01, ***P < 0.001, ****P < 0.0001, ns: not significant.

To confirm the downstream regulatory molecules of miR-155-5p involved in Cur-mediated prevention of DEX-activated PRV, we incubated PRV-infected cells with the miR-155-5P inhibitor and Cur, followed by DEX treatment. The results of the western blotting analysis are shown in Figure 8A, with quantification plots shown in Figures 8B, C, and D. The results from plasmid transfection with Cur for incubation followed by DEX treatment are shown in Figure 8E. Its quantification plots are shown in Figures 8F, G, and H. The results indicated that Aak1 (*P* < 0.0001) (Figures 8A, B, and F) and Notch2 protein levels (*P* < 0.0001) (Figures 8A, C, G) were increased, while the Numb protein level was decreased in the PRV + DEX group compared to the PRV group (*P* < 0.0001) (Figures 8A, D, and H). In the PRV + Cur + DEX group, Aak1 (*P* < 0.0001) (Figures 8A, B, and F) and Notch2 protein levels (*P* < 0.0001) (Figures 8A, C, and G) were decreased, whereas Numb protein levels were increased (*P* < 0.0001) (Figures 8A, D, and H) compared to the PRV + DEX group.

Both Aak1 (*P* < 0.0001) (Figures 8A, B, and F) and Notch2 (*P* < 0.0001) (Figures 8A, C, and G) protein levels were decreased. In contrast, Numb protein (*P* = 0.0055) (Figures 8A, D, and H) levels increased more in the Cur-incubated group than in the Cur-free group following transfection of the miR-155-5P inhibitor in the PRV group. Compared to the PRV group without Cur, the PRV group incubated with Cur after transfection of the Aak1 overexpression plasmid showed decreased protein levels of both Aak1 (*P* < 0.0001) (Figures 8A, B, and F) and Notch2 (*P* < 0.0001) (Figures 8A, C, and G), while Numb protein levels (*P* < 0.0001) (Figures 8A, D, and H) were increased.

These results indicate that Cur, at a concentration of 10 µM, up-regulates miR-155-5P levels in PRV + DEX cells, thus inhibiting Aak1 expression and leading to increased Numb and Notch2 expression. In brief, the miR-155-5p-Aak1-Numb/Notch2 axis mediates the role of Cur in preventing DEX-activated PRV.

## Discussion

The latent PRV infection rate on pig farms is extremely high [45] and is challenging to eliminate. Exposure to external stimuli can easily activate PRV, resulting in a pseudorabies outbreak that poses a serious threat to the pig farming industry. Therefore, preventing stress-induced PRV activation in pig farming is crucial. Notably, our results suggest that Cur prevents DEX from activating PRV in PC-12 cells and that the miR-155-5p-Aak1-Numb/Notch2 axis mediates this process.

During primary herpesvirus infection or activation, the virus causes partial neuronal damage and fragmentation [46]. It disrupts mitochondrial function [38], increasing ROS levels, a by-product of the electron transport chain. As a result of this process, the damaged neurons undergo apoptosis, leading to reduced cell viability. MMP is an important indicator of mitochondrial health and function; therefore, changes in MMP levels are early indicators of cell viability [47]. Cumulatively, the measurement of ROS content, MMP levels, and cell viability can indicate damage to mitochondria and cells, reflecting PRV activity. Our study showed that PRV activity in the cells was inhibited when PC-12 cells were infected with PRV at an MOI of 1 for 24 h. Furthermore, there was no significant increase in the amount of ROS and virions released, which is consistent with previous reports [4]. These findings also align with earlier reports showing that it is difficult for PRV to undergo infectious particle release in neuronal cells in vitro when the MOI is between 0.1 and 1 [48].

Furthermore, viral activation indicators include the replication and transcription of viral genes [49]. PRV protein levels can be monitored using an immunofluorescence assay to demonstrate PRV proliferation [50]. This study confirmed the activation of PRV after incubation with DEX (0.5 µM) for 4 h based on decreased cell viability and MMP levels, increased ROS level, copy number of viral genes, viral load, and PRV protein levels. These results are also consistent with the findings of a previous study [4].

The miRNAs and mRNAs of host cells are reportedly involved in cascade responses to viral infection [51–53]. One previous study identified several DE miRNAs, including miR-155-5p, after the DEX treatment of PRV-infected PC-12 cells using miRNA-Seq techniques [4]. Additionally, a major challenge in the analysis of miRNA function is identifying key targets and targets of action [54]. Nonetheless, our study found that miR-155-5P negatively regulates Aak1 expression during the inhibition and destruction of PRV activity, which is consistent with the negative regulatory profile of miRNAs [54]. Furthermore, the dual-luciferase reporter assay, a decisive method for identifying direct miRNA targets [55], confirmed Aak1 as a functional target of miR-155-5P.

As a member of the NAK family, Aak1 is a significant endocytic kinase that plays an important role in viral infection, assembly, and entry by regulating clathrin-mediated endocytosis through Numb [18, 19, 56]. Furthermore, it is widely known that Numb can negatively regulate the Notch signalling pathways involved in many physiological and pathological processes [57–60]. Studies have also shown that Notch2 is essential in inhibiting herpesvirus activity and the transition of the cleavage cycle [15, 20]. This outcome suggests that the Aak1-Numb-Notch2 signalling pathway in host cells may mediate viral activation or inhibition. Our study found that the overexpression of Aak1 and the miR-155-5p inhibitor significantly increased Aak1 and Notch2 protein levels in PC-12 cells while also significantly decreasing Numb protein levels, which activate PRV. However, conversely, the opposite was true after transfection of miR-155-5p mimics and Aak1 gene-interfering plasmids, suggesting that the miR-155-5p-Aak1-Numb/Notch2 signalling axis mediates the DEX-activated PRV process Table 1.

Notably, the first report on the antiviral effects of Cur on herpes was in 2008 [61], with further studies demonstrating that Cur inhibits EBV reactivation by inhibiting the transcription of the *BZLF1* gene [62]. Similarly, our results suggest that 10 µM Cur up-regulates the levels of miR-155-5P in the DEX-induced PRV-activating group. This process targets the inhibition of Aak1 expression, increasing Numb levels and decreasing Notch2 levels. However, this outcome was reversed when cells were transfected with a miR-155-5p inhibitor and *Aak1* gene overexpression plasmids, suggesting that the miR-155-5p-Aak1-Numb/Notch2 axis mediates the process by which Cur prevents PRV activation, providing a crucial foundation for the clinical application of Cur. Furthermore, recent research has shown that miR-155-5p can regulate the proliferation of HSV-1, which shares similar latency and reactivation properties with PRV [63, 64], and Notch regulates 2BHV-1 during latency [65, 66]. These results suggest that our findings could apply to other viruses with similar reactivation mechanisms. However, while Numb can negatively regulate the Notch signalling pathway involved in many physiological and pathological processes [57–60], further studies are needed to determine whether it can negatively regulate Notch in the process of Cur prevention in DEX-activated PRV. In addition, the upstream regulatory factors that modulate miR-155-5p expression after DEX treatment need further exploration, and our findings need to be validated through in vivo studies.

In this study, we found that the miR-155-5p/Aak1-Numb/Notch2 axis mediated the inhibition of DEX-activated PRV in PC-12 cells. A concentration of 10 µM Cur prevented DEX-induced PRV activation by up-regulating miR-155-5p levels in the PRV-activated group, which targeted and inhibited Aak1 expression, resulting in increased Numb protein levels and a decrease in Notch2 protein levels. To our knowledge, this is the first report on the role of Cur in preventing DEX-induced activation of PRV through the miR-155-5p-Aak1-Numb/Notch2 pathway in neural cells. Our findings provide new insights into the mechanisms underlying glucocorticoid-induced PRV activation and the beneficial effects of Cur on glucocorticoid-activated PRV.

## Supplementary Information

**Additional file 1:** **Virus content in PC-12 cell culture supernatant of different treatment groups.** A: PK-15 was incubated with PC-12 cell culture supernatant infected with PRV at an MOI of 1 for 24 h. B: PK-15 was incubated with the supernatant of PC-12 culture without PRV infection. C: PC-12 cells were incubated with DEX (0.5 µM), and the supernatant was incubated with PK-15 for 4h. D: PK-15 was incubated with the supernatant of PC-12 cells after infection with PRV at an MOI of 1 for 24h and incubated with DEX (0.5 µM) for 4 h.

**Additional file 2:** **Relative mRNA levels of Wee1 and CEBPB genes in PC-12 cells of different treatment groups.** A: Wee1 gene (η² = 0.953). B: CEBPB (η² = 0.910). Mean ± SD, *n* = 3, *P < 0.05, **P < 0.01, ***P < 0.001, ****P < 0.0001, ns: not significant. CON: blank control; DEX:4 h incubation with 0.5µM DEX; PRV: PRV infection with MOI=1 for 24 h; PRV+DEX: PRV with MOI=1 was infected for 24 h and then incubation with 0.5µM DEX for 4 h.

**Additional file 3:** **Effect of miR-155-5p mimic/inhibitor transfection on miR-155-5p, Wee1 and CEBPB mRNA levels.** A: miR-155-5p (η² = 0.934). B: Wee1 (η² = 0.594). C: CEBPB (η² = 0.848). Mean ± SD, *n* = 3, *P < 0.05, **P < 0.01, ***P < 0.001, ****P < 0.0001, ns: not significant.

**Additional file 4:** **Construction of recombinant plasmid.** A: Binding site. B: GP-miRGLO-miR-155-5p-Aak1-WT recombinant plasmid sequence. C: The GP-miRGLO-miR-155-5p-Aak1-MUT recombinant plasmid sequence. D: Enzymatic digestion of the recombinant plasmid.

**Additional file 5:** **Transfection effect of Aak1 overexpression plasmid.** A: Transfection effect observed under a fluorescence microscope (10 × 10 magnification). B: Aak1 mRNA levels (η² = 0.987). C: Aak1 protein levels (η² = 0.995). Mean ± SD, *n* = 3, *P < 0.05, **P < 0.01, ***P < 0.001, ****P < 0.0001, ns: not significant.

**Additional file 6:** **Transfection effect of Aak1 interference plasmid.** A: Transfection effect of shNC observed under a fluorescence microscope (10 × 10). B: Inhibitory effect of the shRNA-positive control (η² = 0.981). C: Effects of shRNA transfection on Aak1 mRNA levels (η² = 0.989). D: Effects of shRNA transfection on Aak1 protein levels (η² = 0.944). Mean ± SD, *n* = 3, *P < 0.05, **P < 0.01, ***P < 0.001, ****P < 0.0001, ns: not significant.

**Additional file 7:** **Toxicity of different concentrations of Cur on PC-12 cells (η² = 0.805).** Mean ± SD, *n* = 3, *P < 0.05, **P < 0.01, ***P < 0.001, ****P < 0.0001, ns: not significant.

**Additional file 8:** **The concentration of Cur inhibited the virion content in the supernatant of DEX-activated PRV cells.** A: PRV with an MOI of 100, directly infected with PK-15. B: PK-15 was infected with the supernatant of PC-12 cells after 24 h of PRV infection with MOI=1 and incubation with 0.5 µM DEX for 4 h. C: PC-12 cells without virus infection were incubated with 0.5 µM DEX for 4 h, and PK-15 was incubated with the culture supernatant. D: PRV with MOI=1 directly infects PK-15. E: PK-15 cells were incubated directly with 5 µM Cur. F: PRV with MOI=1 infected PC-12 cells for 24 h+5 µM Cur incubated PC-12 cells for 24 h+0.5 µM DEX incubated PC-12 cells for 4 h and cultured supernatant incubated PK-15 cells. G: PK-15 cells were incubated directly with 0.5 µM DEX. H: PK-15 cells were infected with PC-12 cell supernatant 24 h after direct incubation of 0.5 µM DEX with PK-MOI=1 PRV. I: PK-15 cells were incubated directly with 10 µM Cur. J: PRV with MOI=1 infected PC-12 cells for 24 h+10 µM Cur incubated PC-12 cells for 24 h+0.5 µM DEX incubated PC-12 cells for 4 h and cultured supernatant incubated PK-15 cells. K: PK-15 cells were incubated with the supernatants of PC-12 cells without viral infection. L: Blank control.

## Data Availability

All data generated or analyzed during this study are included in this published article. Further inquiries can be directed to the corresponding authors.

## Abbreviations

PRV: pseudorabies virus
Cur: curcumin
MOI: multiplicity of infection
DEX: dexamethasone
mRNA: messenger RNA
miR-155-5p: microRNA-155-5p
Aak1: AP2 associated kinase 1
Numb: NUMB endocytic adaptor protein
Notch2: Notch receptor 2
PR: pseudorabies
miRNAs: microRNAs
ATP5B: ATP synthase subunit beta
HSV-1: herpesvirus-1
CME: clathrin-mediated endocytosis
EBV: Epstein-Barr virus
MMP: mitochondrial membrane potential
ROS: reactive oxygen species
IE180: immediate-early 180 gene
RT-qPCR: quantitative real-time PCR
Wee1: WEE1 G2 checkpoint kinas
CEBPB: CCAAT enhancer binding protein beta
shRNA: short hairpin RNA
DMEM: Dulbecco’s modified Eagle’s medium
FBS: foetal bovine serum
SD: standard deviation
ANOVA: analysis of variance

## References

[1] Verpoest S, Redant V, Cay AB, Favoreel H, De Regge N (2018) Reduced virulence of a pseudorabies virus isolate from wild boar origin in domestic pigs correlates with hampered visceral spread and age-dependent reduced neuroinvasive capacity. Virulence 9:149–162 28873002 10.1080/21505594.2017.1368941 PMC5955469

[2] Wang HH, Liu J, Li LT, Chen HC, Zhang WP, Liu ZF (2020) Typical gene expression profile of pseudorabies virus reactivation from latency in swine trigeminal ganglion. J Neurovirol 26:687–695 32671812 10.1007/s13365-020-00866-9

[3] Santos VC, Ostler JB, Harrison KS, Jones C (2023) Slug, a stress-induced transcription factor, stimulates herpes simplex virus 1 replication and transactivates a cis-regulatory module within the VP16 promoter. J Virol 97:e0007323 37022165 10.1128/jvi.00073-23 PMC10134811

[4] Zhang C, Liu Y, Yang F, Liu Y, Wang N, Li Y, Liu Y, Qiu Z, Zhang L, You X, Gan L (2024) MicroRNA-194-5p/heparin-binding EGF-like growth factor signaling mediates dexamethasone-induced activation of pseudorabies virus in rat pheochromocytoma cells. Vet Microbiol 290:109974 38262115 10.1016/j.vetmic.2023.109974

[5] Yazdi IK, Taghipour N, Hmaidan S, Palomba R, Scaria S, Munoz A, Boone TB, Tasciotti E (2016) Antibody-mediated inhibition of Nogo-A signaling promotes neurite growth in PC-12 cells. J Tissue Eng 7:2041731416629767 27027860 10.1177/2041731416629767 PMC4794088

[6] Hsiao YS, Lin CC, Hsieh HJ, Tsai SM, Kuo CW, Chu CW, Chen P (2011) Manipulating location, polarity, and outgrowth length of neuron-like pheochromocytoma (PC-12) cells on patterned organic electrode arrays. Lab Chip 11:3674–3680 21922117 10.1039/c1lc20675c

[7] Gao Y, Hu JH, Liang XD, Chen J, Liu CC, Liu YY, Cheng Y, Go YY, Zhou B (2021) Curcumin inhibits classical swine fever virus replication by interfering with lipid metabolism. Vet Microbiol 259:109152 34146894 10.1016/j.vetmic.2021.109152

[8] Workman A, Eudy J, Smith L, da Silva LF, Sinani D, Bricker H, Cook E, Doster A, Jones C (2012) Cellular transcription factors induced in trigeminal ganglia during dexamethasone-induced reactivation from latency stimulate bovine herpesvirus 1 productive infection and certain viral promoters. J Virol 86:2459–2473 22190728 10.1128/JVI.06143-11 PMC3302277

[9] Yang EV, Webster Marketon JI, Chen M, Lo KW, Kim SJ, Glaser R (2010) Glucocorticoids activate Epstein Barr virus lytic replication through the upregulation of immediate early BZLF1 gene expression. Brain Behav Immun 24:1089–1096 20466055 10.1016/j.bbi.2010.04.013 PMC2939213

[10] Zhao J, Zhu L, Wijesekera N, Jones C (2020) Specific Akt family members impair stress-mediated transactivation of viral promoters and enhance neuronal differentiation: important functions for maintaining latency. J Virol 94:e00901-20 32796067 10.1128/JVI.00901-20 PMC7565622

[11] Djuranovic S, Nahvi A, Green R (2012) miRNA-mediated gene silencing by translational repression followed by mRNA deadenylation and decay. Science 336:237–240 22499947 10.1126/science.1215691 PMC3971879

[12] Fan Y, Zhu L, Sun X, Lyu W, Xu L, Yin Y, Zhao J, Huang J, Den Y, Jiang Z, Xu S, Mao X, Xu Z (2019) Exploring the tissue tropism of pseudorabies virus based on miRNA level analysis. BMC Microbiol 19:125 31185898 10.1186/s12866-019-1497-4 PMC6558711

[13] Zheng SQ, Li YX, Zhang Y, Li X, Tang H (2011) MiR-101 regulates HSV-1 replication by targeting ATP5B. Antivir Res 89:219–226 21291913 10.1016/j.antiviral.2011.01.008

[14] Lagos D, Pollara G, Henderson S, Gratrix F, Fabani M, Milne RS, Gotch F, Boshoff C (2010) miR-132 regulates antiviral innate immunity through suppression of the p300 transcriptional co-activator. Nat Cell Biol 12:513–519 20418869 10.1038/ncb2054

[15] Giunco S, Celeghin A, Gianesin K, Dolcetti R, Indraccolo S, De Rossi A (2015) Cross talk between EBV and telomerase: the role of TERT and NOTCH2 in the switch of latent/lytic cycle of the virus. Cell Death Dis 6:e1774 26018735 10.1038/cddis.2015.145 PMC4669716

[16] Karim M, Saul S, Ghita L, Sahoo MK, Ye C, Bhalla N, Lo CW, Jin J, Park JG, Martinez-Gualda B, East MP, Johnson GL, Pinsky BA, Martinez-Sobrido L, Asquith CRM, Narayanan A, De Jonghe S, Einav S (2022) Numb-associated kinases are required for SARS-CoV-2 infection and are cellular targets for antiviral strategies. Antivir Res 204:105367 35738348 10.1016/j.antiviral.2022.105367 PMC9212491

[17] Oh DS, Park JH, Jung HE, Kim HJ, Lee HK (2021) Autophagic protein ATG5 controls antiviral immunity via glycolytic reprogramming of dendritic cells against respiratory syncytial virus infection. Autophagy 17:2111–2127 32816604 10.1080/15548627.2020.1812218 PMC8496528

[18] Pu S, Schor S, Karim M, Saul S, Robinson M, Kumar S, Prugar LI, Dorosky DE, Brannan J, Dye JM, Einav S (2020) BIKE regulates dengue virus infection and is a cellular target for broad-spectrum antivirals. Antivir Res 184:104966 33137362 10.1016/j.antiviral.2020.104966 PMC7879702

[19] Tongmuang N, Yasamut U, Noisakran S, Sreekanth GP, Yenchitsomanus PT, Limjindaporn T (2020) Suppression of µ1 subunit of the adaptor protein complex 2 reduces dengue virus release. Virus Genes 56:27–36 31720911 10.1007/s11262-019-01710-x

[20] Rowe M, Raithatha S, Shannon-Lowe C (2014) Counteracting effects of cellular Notch and Epstein-Barr virus EBNA2: implications for stromal effects on virus-host interactions. J Virol 88:12065–12076 25122803 10.1128/JVI.01431-14 PMC4178707

[21] Wang C, Wang J, Shuai L, Ma X, Zhang H, Liu R, Chen W, Wang X, Ge J, Wen Z, Bu Z (2019) The serine/threonine kinase AP2-associated kinase 1 plays an important role in rabies virus entry. Viruses 12:45 31905947 10.3390/v12010045 PMC7019586

[22] Conner SD, Schmid SL (2002) Identification of an adaptor-associated kinase, AAK1, as a regulator of clathrin-mediated endocytosis. J Cell Biol 156:921–929 11877461 10.1083/jcb.200108123 PMC2173317

[23] Henderson DM, Conner SD (2007) A novel AAK1 splice variant functions at multiple steps of the endocytic pathway. Mol Biol Cell 18:2698–2706 17494869 10.1091/mbc.E06-09-0831 PMC1924820

[24] Conner SD, Schröter T, Schmid SL (2003) AAK1-mediated micro2 phosphorylation is stimulated by assembled clathrin. Traffic 4:885–890 14617351 10.1046/j.1398-9219.2003.0142.x

[25] Smythe E, Ayscough KR (2003) The Ark1/Prk1 family of protein kinases. regulators of endocytosis and the actin skeleton. EMBO Rep 4:246–251 12634840 10.1038/sj.embor.embor776 PMC1315904

[26] Marton LT, Pescinini-E-Salzedas LM, Camargo MEC, Barbalho SM, Haber JFDS, Sinatora RV, Detregiachi CRP, Girio RJS, Buchaim DV, Dos Santos C, Bueno P (2021) The effects of curcumin on diabetes mellitus: a systematic review. Front Endocrinol 12:669448 10.3389/fendo.2021.669448 PMC8126655 34012421

[27] Tomeh MA, Hadianamrei R, Zhao X (2019) A review of curcumin and its derivatives as anticancer agents. Int J Mol Sci 20:1033 30818786 10.3390/ijms20051033 PMC6429287

[28] Fu YS, Chen TH, Weng L, Huang L, Lai D, Weng CF (2021) Pharmacological properties and underlying mechanisms of curcumin and prospects in medicinal potential. Biomed Pharmacother 141:111888 34237598 10.1016/j.biopha.2021.111888

[29] Zahra M, Mostafa T, Yasaman H, Amin D, Mostafa S, Seyed Sina N, Sheida J, Elnaz R (2022) Review on the effects curcumin on tumors of the reproductive system: curcumin and reproductive tumors. Galen Med J 10:1–8 35855104 10.31661/gmj.v10i0.2178 PMC9261988

[30] Fang JH, Chiu TL, Huang WC, Lai YH, Hu SH, Chen YY, Chen SY (2016) Dual-targeting lactoferrin-conjugated polymerized magnetic polydiacetylene-assembled nanocarriers with self-responsive fluorescence/magnetic resonance imaging for in vivo brain tumor therapy. Adv Healthc Mater 5:688–695 26820074 10.1002/adhm.201500750

[31] Man S, Liu W, Bi J, Bai J, Wu Q, Hu B, Hu J, Ma L (2024) Smart mesoporous silica nanoparticles loading curcumin inhibit liver cancer. J Agric Food Chem 72:25743–25754 39506560 10.1021/acs.jafc.4c08202

[32] Cheng B, Lin J, Zou J, Zhuang Y, Zheng L, Zhang G, Huang B, Fei P (2024) Preparation of curcumin-loaded pectin-nisin copolymer emulsion and evaluation of its stability. Int J Biol Macromol 254:127812 37923038 10.1016/j.ijbiomac.2023.127812

[33] Sharma RA, Steward WP, Gescher AJ (2007) Pharmacokinetics and pharmacodynamics of curcumin. Adv Exp Med Biol 595:453–470 17569224 10.1007/978-0-387-46401-5_20

[34] Ravindranath V, Chandrasekhara N (1981) Metabolism of curcumin–studies with [3H] curcumin. Toxicology 22:337–344 7342372 10.1016/0300-483x(81)90027-5

[35] Orellana-Paucar AM, Machado-Orellana MG (2022) Pharmacological profile, bioactivities, and safety of turmeric oil. Molecules 27:5055 36014301 10.3390/molecules27165055 PMC9414992

[36] ELBini-Dhouib I, Doghri R, Ellefi A, Degrach I, Srairi-Abid N, Gati A (2021) Curcumin Attenuated neurotoxicity in sporadic animal model of Alzheimer’s disease. Molecules 26:3011 34070220 10.3390/molecules26103011 PMC8158738

[37] Butnariu M, Quispe C, Koirala N, Khadka S, Salgado-Castillo CM, Akram M, Anum R, Yeskaliyeva B, Cruz-Martins N, Martorell M, Kumar M, Vasile Bagiu R, Abdull Razis AF, Sunusi U, Muhammad Kamal R, Sharifi-Rad J (2022) Bioactive effects of curcumin in human immunodeficiency virus infection along with the most effective isolation techniques and type of nanoformulations. Int J Nanomed 17:3619–3632 10.2147/IJN.S364501 PMC9391931 35996526

[38] Yang B, Luo G, Zhang C, Feng L, Luo X, Gan L (2020) Curcumin protects rat hippocampal neurons against pseudorabies virus by regulating the BDNF/TrkB pathway. Sci Rep 10:22204 33335121 10.1038/s41598-020-78903-0 PMC7746732

[39] Hl DU, He DQ, Wang JL, Che YF, Yan QH (2022) Curcumin targeting miR-155-5p/TP53INP1 axis induced oxidative stress to regulate salivary gland tumor cell proliferation and apoptosis. Shanghai Kou Qiang Yi Xue 31:483–490 36758595

[40] Pritchard CC, Cheng HH, Tewari M (2012) MicroRNA profiling: approaches and considerations. Nat Rev Genet 13:358–369 22510765 10.1038/nrg3198 PMC4517822

[41] Kumar A, Wong AK, Tizard ML, Moore RJ, Lefèvre C (2012) miRNA_Targets: a database for miRNA target predictions in coding and non-coding regions of mRNAs. Genomics 100:352–356 22940442 10.1016/j.ygeno.2012.08.006

[42] Mendonça LM, da Silva MC, Teixeira CC, de Freitas LA, MdeL B, Antunes LM (2013) Curcumin reduces cisplatin-induced neurotoxicity in NGF-differentiated PC12 cells. Neurotoxicology 34:205–211 23036615 10.1016/j.neuro.2012.09.011

[43] Rahaman MS, Banik S, Akter M, Rahman MM, Sikder MT, Hosokawa T, Saito T, Kurasaki M (2020) Curcumin alleviates arsenic-induced toxicity in PC12 cells via modulating autophagy/apoptosis. Ecotoxicol Environ Saf 200:110756 32464442 10.1016/j.ecoenv.2020.110756

[44] Zarei M, Esmaeili A, Zarrabi A, Zarepour A (2022) superparamagnetic iron oxide nanoparticles and curcumin equally promote neuronal branching morphogenesis in the absence of nerve growth factor in PC12 cells. Pharmaceutics 14:2692 36559186 10.3390/pharmaceutics14122692 PMC9788162

[45] Pomeranz LE, Reynolds AE, Hengartner CJ (2005) Molecular biology of pseudorabies virus: impact on neurovirology and veterinary medicine. Microbiol Mol Biol Rev 69:462–500 16148307 10.1128/MMBR.69.3.462-500.2005 PMC1197806

[46] Doll JR, Hoebe K, Thompson RL, Sawtell NM (2020) Resolution of herpes simplex virus reactivation in vivo results in neuronal destruction. PLoS Pathog 16:e1008296 32134994 10.1371/journal.ppat.1008296 PMC7058292

[47] Perelman A, Wachtel C, Cohen M, Haupt S, Shapiro H, Tzur A (2012) JC-1: alternative excitation wavelengths facilitate mitochondrial membrane potential cytometry. Cell Death Dis 3:e430 23171850 10.1038/cddis.2012.171 PMC3542606

[48] Koyuncu OO, Song R, Greco TM, Cristea IM, Enquist LW (2015) The number of alphaherpesvirus particles infecting axons and the axonal protein repertoire determines the outcome of neuronal infection. MBio 6:e00276-15 25805728 10.1128/mBio.00276-15 PMC4453538

[49] Abere B, Samarina N, Gramolelli S, Rückert J, Gerold G, Pich A, Schulz TF (2018) Kaposi’s sarcoma-associated herpesvirus nonstructural membrane protein pK15 recruits the class II phosphatidylinositol 3-kinase PI3K-C2α to activate productive viral replication. J Virol 92:e00544-18 29950425 10.1128/JVI.00544-18 PMC6096797

[50] Yang S, Pei Y, Zhao A (2017) iTRAQ-based proteomic analysis of porcine kidney epithelial PK15 cells infected with pseudorabies virus. Sci Rep 7:45922 28374783 10.1038/srep45922 PMC5379687

[51] Fu YR, Liu XJ, Li XJ, Shen ZZ, Yang B, Wu CC, Li JF, Miao LF, Ye HQ, Qiao GH, Rayner S, Chavanas S, Davrinche C, Britt WJ, Tang Q, McVoy M, Mocarski E, Luo MH (2015) MicroRNA miR-21 attenuates human cytomegalovirus replication in neural cells by targeting Cdc25a. J Virol 89:1070–1082 25378484 10.1128/JVI.01740-14 PMC4300626

[52] Hill JM, Zhao Y, Clement C, Neumann DM, Lukiw WJ (2009) HSV-1 infection of human brain cells induces miRNA-146a and Alzheimer-type inflammatory signaling. NeuroReport 20:1500–1505 19801956 10.1097/WNR.0b013e3283329c05 PMC2872932

[53] Sun B, Yang X, Hou F, Yu X, Wang Q, Oh HS, Raja P, Pesola JM, Vanni EAH, McCarron S, Morris-Love J, Ng AHM, Church GM, Knipe DM, Coen DM, Pan D (2021) Regulation of host and virus genes by neuronal miR-138 favours herpes simplex virus 1 latency. Nat Microbiol 6:682–696 33558653 10.1038/s41564-020-00860-1 PMC8221016

[54] Eulalio A, Mano M (2015) MicroRNA screening and the quest for biologically relevant targets. J Biomol Screen 20:1003–1017 25824005 10.1177/1087057115578837

[55] Bartel DP (2004) MicroRNAs: genomics, biogenesis, mechanism, and function. Cell 116:281–297 14744438 10.1016/s0092-8674(04)00045-5

[56] Pu SY, Xiao F, Schor S, Bekerman E, Zanini F, Barouch-Bentov R, Nagamine CM, Einav S (2018) Feasibility and biological rationale of repurposing sunitinib and erlotinib for dengue treatment. Antivir Res 155:67–75 29753658 10.1016/j.antiviral.2018.05.001 PMC6064211

[57] Yang J, Bücker S, Jungblut B, Böttger T, Cinnamon Y, Tchorz J, Müller M, Bettler B, Harvey R, Sun QY, Schneider A, Braun T (2012) Inhibition of Notch2 by Numb/Numblike controls myocardial compaction in the heart. Cardiovasc Res 96:276–285 22865640 10.1093/cvr/cvs250

[58] Belle VA, McDermott N, Meunier A, Marignol L (2014) NUMB inhibition of NOTCH signalling as a therapeutic target in prostate cancer. Nat Rev Urol 11:499–507 25134838 10.1038/nrurol.2014.195 PMC5240474

[59] Yan R, Dai W, Wu R, Huang H, Shu M (2022) Therapeutic targeting m6A-guided miR-146a-5p signaling contributes to the melittin-induced selective suppression of bladder cancer. Cancer Lett 534:215615 35278613 10.1016/j.canlet.2022.215615

[60] Benamar M, Chen Q, Chou J, Julé AM, Boudra R, Contini P, Crestani E, Lai PS, Wang M, Fong J, Rockwitz S, Lee P, Chan TMF, Altun EZ, Kepenekli E, Karakoc-Aydiner E, Ozen A, Boran P, Aygun F, Onal P, Sakalli AAK, Cokugras H, Gelmez MY, Oktelik FB, Cetin EA, Zhong Y, Taylor ML, Irby K, Halasa NB, Mack EH, et al. (2023) The Notch1/CD22 signaling axis disrupts Treg function in SARS-CoV-2-associated multisystem inflammatory syndrome in children. J Clin Invest 133:e163235 36282598 10.1172/JCI163235 PMC9797337

[61] Kutluay SB, Doroghazi J, Roemer ME, Triezenberg SJ (2008) Curcumin inhibits herpes simplex virus immediate-early gene expression by a mechanism independent of p300/CBP histone acetyltransferase activity. Virology 373:239–247 18191976 10.1016/j.virol.2007.11.028 PMC2668156

[62] Hassanpour M, Tazarghi A, Teimoori A, Tabaraei A, Erfani-Moghadam V, Yamchi A, Akhondi S, Nikoo HR (2022) Curcumin inhibits the replication of rotavirus in vitro. Acta Virol 66:166–171 35766473 10.4149/av_2022_206

[63] Marcocci ME, Napoletani G, Protto V, Kolesova O, Piacentini R, Li Puma DD, Lomonte P, Grassi C, Palamara AT, De Chiara G (2020) Herpes simplex virus-1 in the brain: the dark side of a sneaky infection. Trends Microbiol 28:808–820 32386801 10.1016/j.tim.2020.03.003

[64] Wang Z, Li K, Wang X, Huang W (2019) MiR-155-5p modulates HSV-1 replication via the epigenetic regulation of SRSF2 gene expression. Epigenetics 14:494–503 30950329 10.1080/15592294.2019.1600388 PMC6557561

[65] Jones C (2003) Herpes simplex virus type 1 and bovine herpesvirus 1 latency. Clin Microbiol Rev 16:79–95 12525426 10.1128/CMR.16.1.79-95.2003 PMC145298

[66] Liu Y, Jones C (2016) Regulation of Notch-mediated transcription by a bovine herpesvirus 1 encoded protein (ORF2) that is expressed in latently infected sensory neurons. J Neurovirol 22:518–528 26846632 10.1007/s13365-015-0394-3

