# Supplementary figures and images for: Curcumin prevents dexamethasone-induced activation of the pseudorabies virus in rat pheochromocytoma cells through the miR-155-5p-Aak1-Numb/Notch2 signalling axis
Source: Vet Res. 2025 Apr 21;56:86. doi: 10.1186/s13567-025-01509-9 (PMC12010530; doi:10.1186/s13567-025-01509-9)

## Slide 1
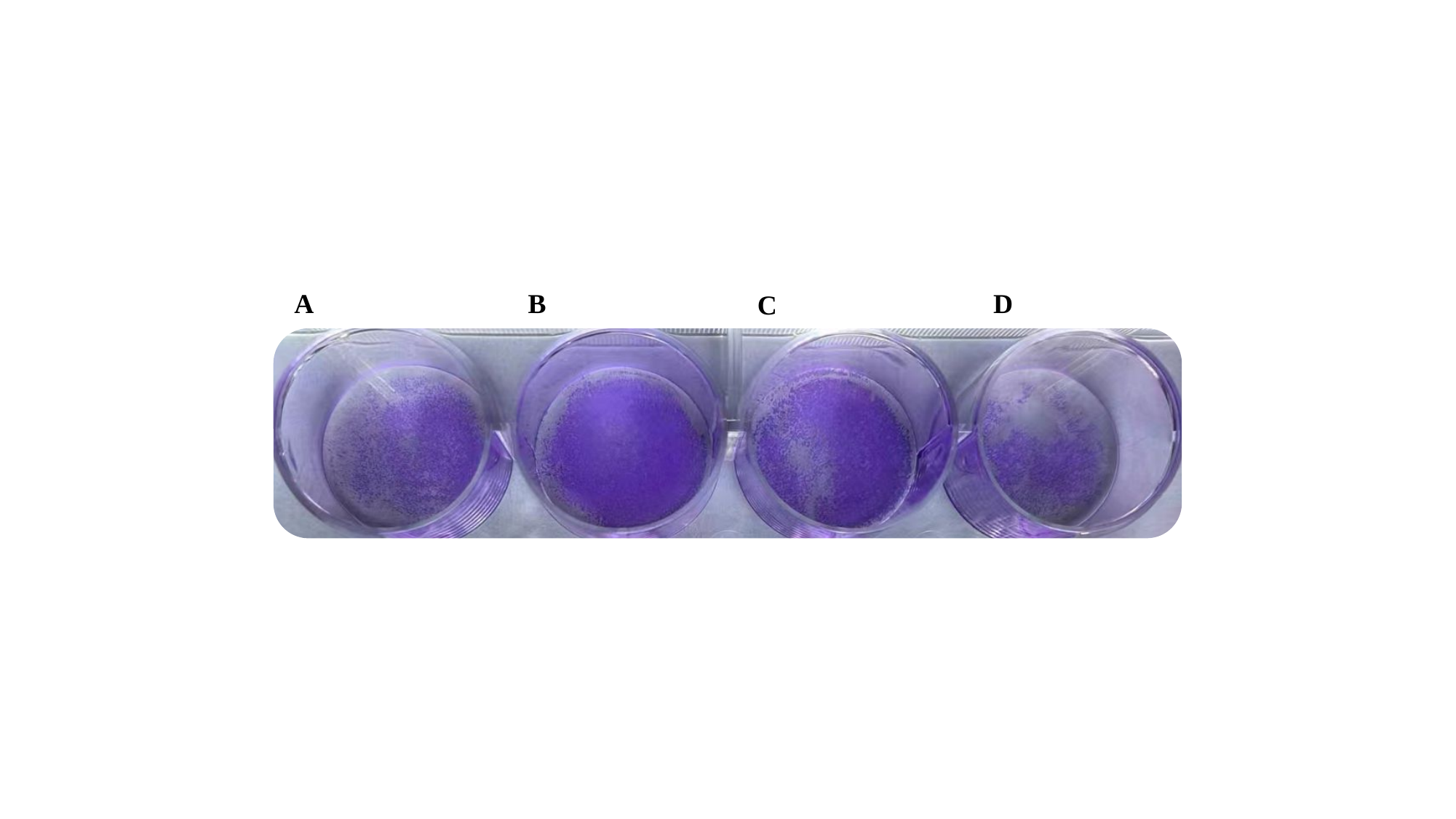

A
B
D
C

Supplement: Supplementary file 1 — Additional file 1: Virus content in PC-12 cell culture supernatant of different treatment groups. A: PK-15 was incubated with PC-12 cell culture supernatant infected with PRV at an MOI of 1 for 24 h. B: PK-15 was incubated with the supernatant of PC-12 culture without PRV infection. C: PC-12 cells were incubated with DEX (0.5 µM), and the supernatant was incubated with PK-15 for 4h. D: PK-15 was incubated with the supernatant of PC-12 cells after infection with PRV at an MOI of 1 for 24h and incubated with DEX (0.5 µM) for 4 h. [file 13567_2025_1509_MOESM1_ESM.pptx]

## Slide 1
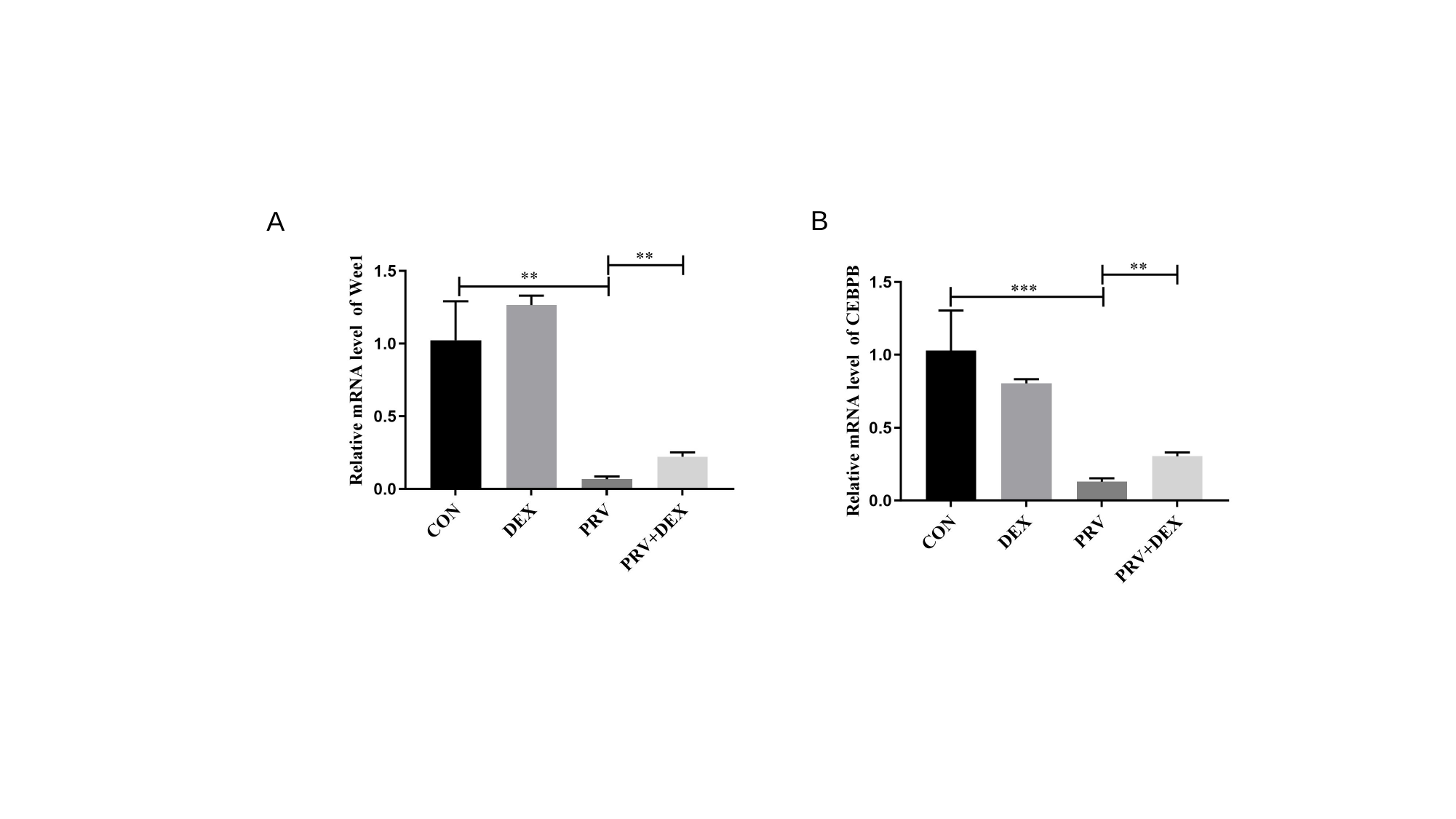

B
A

Supplement: Supplementary file 2 — Additional file 2: Relative mRNA levels of Wee1 and CEBPB genes in PC-12 cells of different treatment groups. A: Wee1 gene (η² = 0.953). B: CEBPB (η² = 0.910). Mean ± SD, n = 3, *P < 0.05, **P < 0.01, ***P < 0.001, ****P < 0.0001, ns: not significant. CON: blank control; DEX:4 h incubation with 0.5µM DEX; PRV: PRV infection with MOI=1 for 24 h; PRV+DEX: PRV with MOI=1 was infected for 24 h and then incubation with 0.5µM DEX for 4 h. [file 13567_2025_1509_MOESM2_ESM.pptx]

## Slide 1
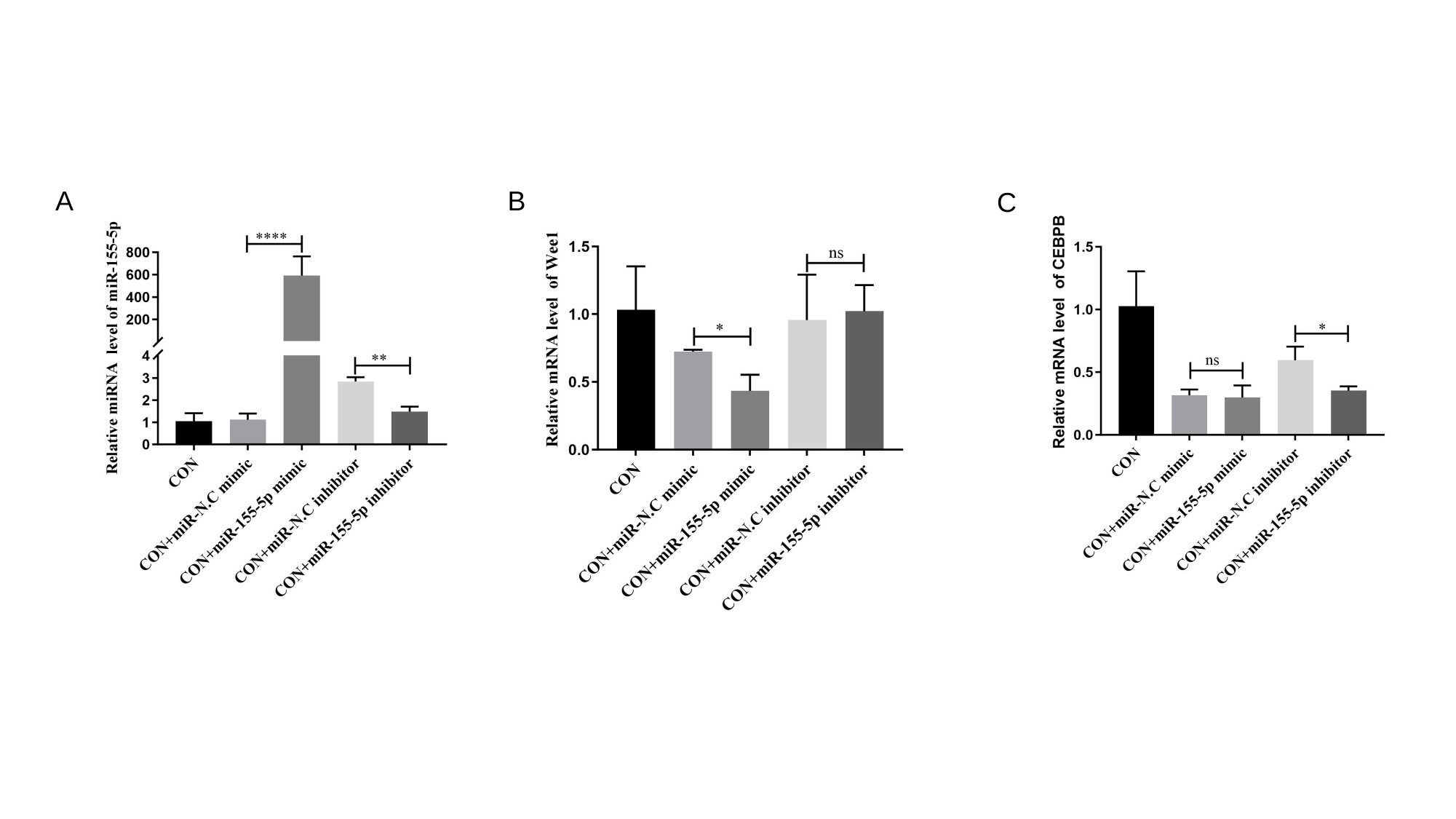

A
B
C

Supplement: Supplementary file 3 — Additional file 3: Effect of miR-155-5p mimic/inhibitor transfection on miR-155-5p, Wee1 and CEBPB mRNA levels. A: miR-155-5p (η² = 0.934). B: Wee1 (η² = 0.594). C: CEBPB (η² = 0.848). Mean ± SD, n = 3, *P < 0.05, **P < 0.01, ***P < 0.001, ****P < 0.0001, ns: not significant. [file 13567_2025_1509_MOESM3_ESM.pptx]

## Slide 1
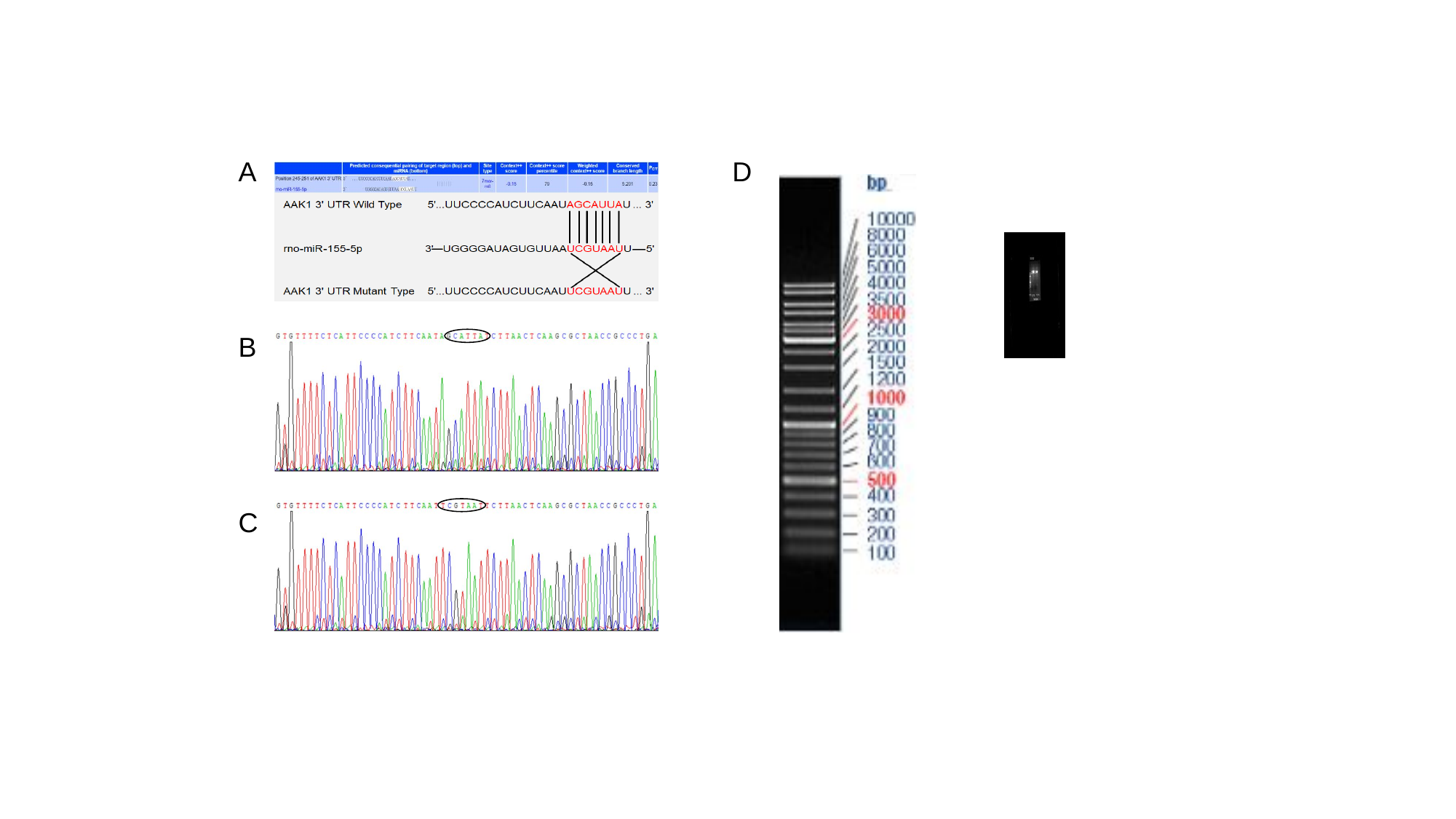

A
D
B
C

Supplement: Supplementary file 4 — Additional file 4: Construction of recombinant plasmid. A: Binding site. B: GP-miRGLO-miR-155-5p-Aak1-WT recombinant plasmid sequence. C: The GP-miRGLO-miR-155-5p-Aak1-MUT recombinant plasmid sequence. D: Enzymatic digestion of the recombinant plasmid. [file 13567_2025_1509_MOESM4_ESM.pptx]

## Slide 1
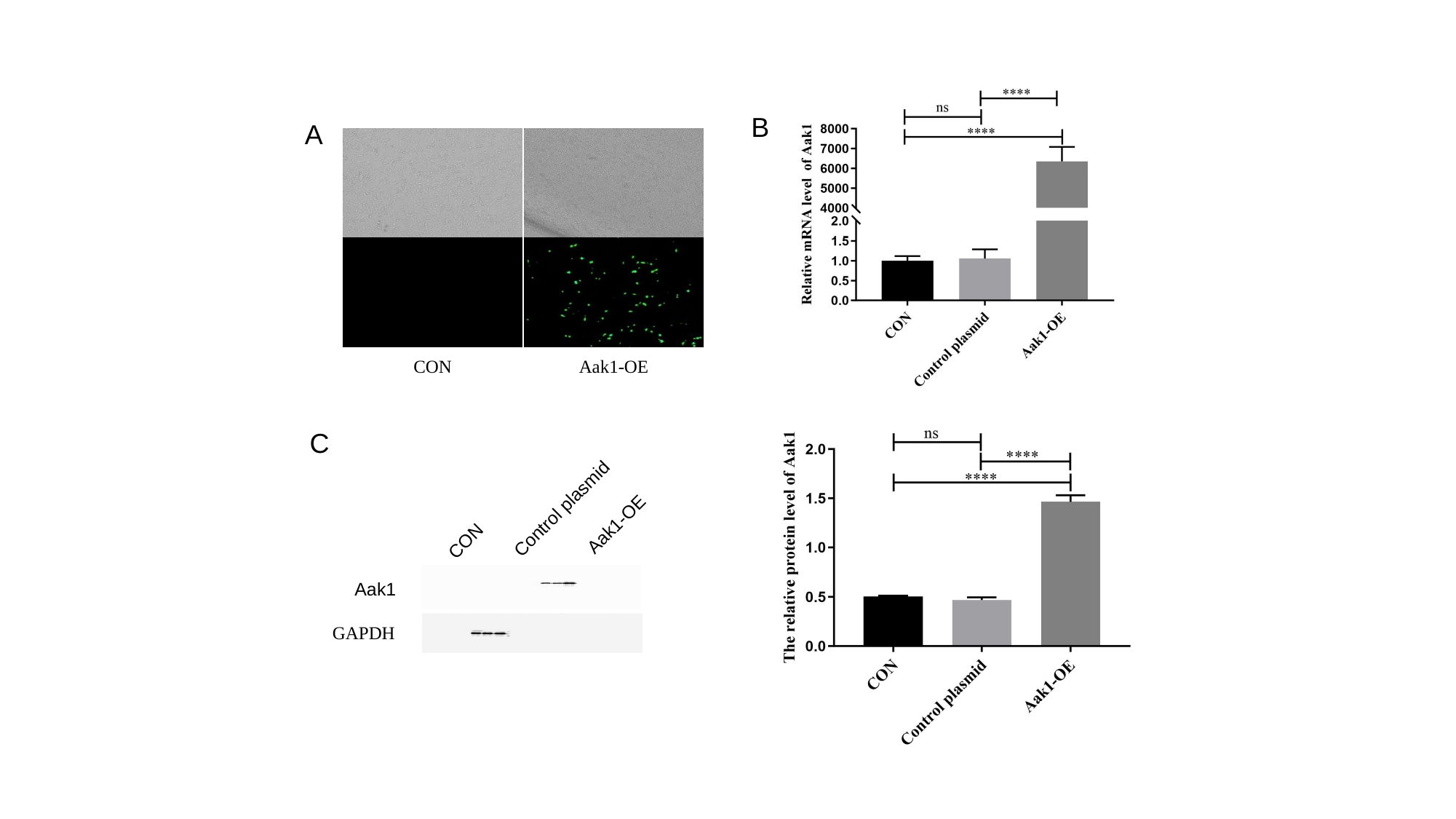

B
A
CON
Aak1-OE
C
Control plasmid
Aak1-OE
CON
Aak1
GAPDH

Supplement: Supplementary file 5 — Additional file 5: Transfection effect of Aak1 overexpression plasmid. A: Transfection effect observed under a fluorescence microscope (10 × 10 magnification). B: Aak1 mRNA levels (η² = 0.987). C: Aak1 protein levels (η² = 0.995). Mean ± SD, n = 3, *P < 0.05, **P < 0.01, ***P < 0.001, ****P < 0.0001, ns: not significant. [file 13567_2025_1509_MOESM5_ESM.pptx]

## Slide 1
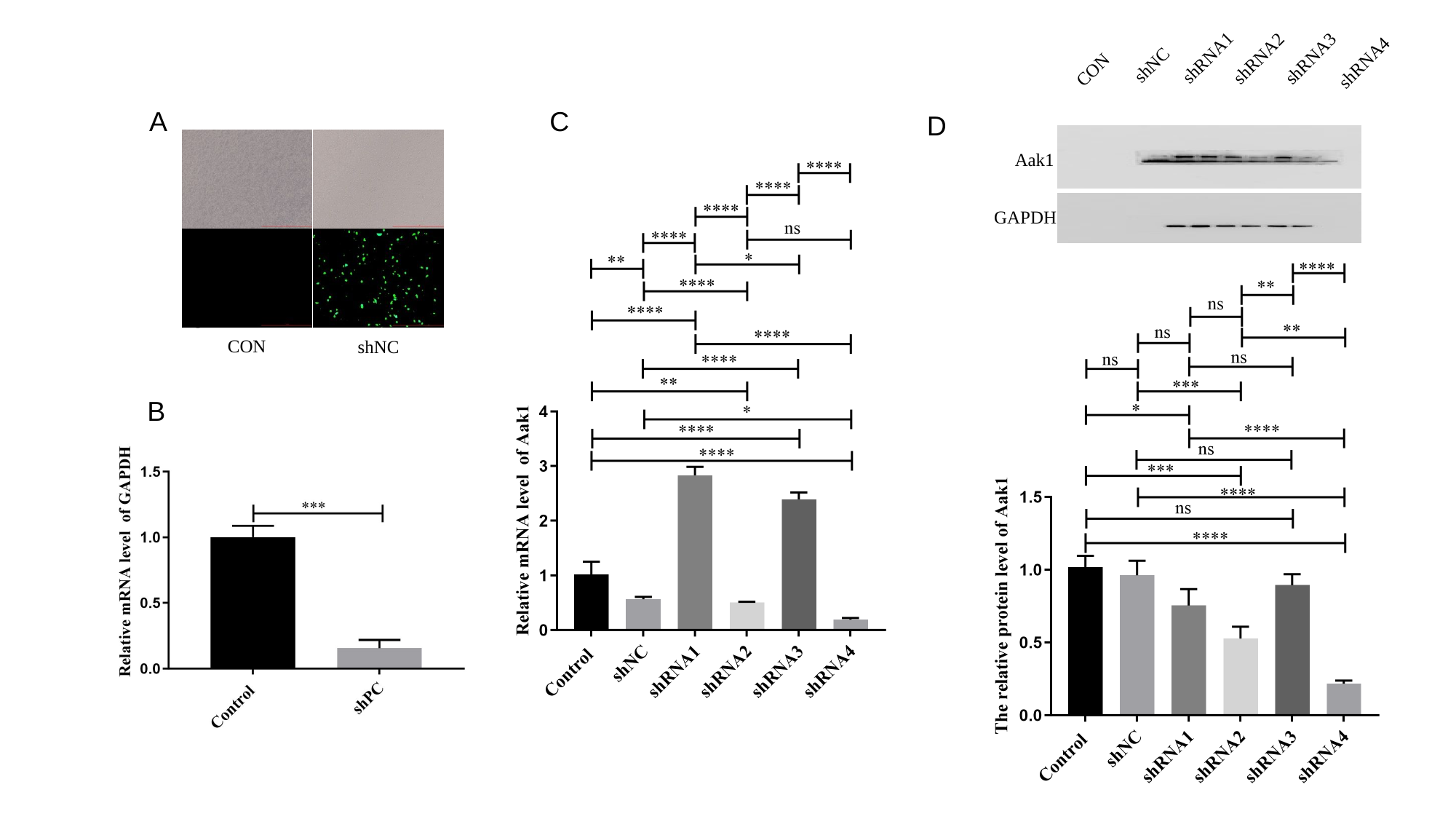

shRNA3
shRNA1
shRNA4
shRNA2
CON
shNC
Aak1
GAPDH
A
C
D
C
CON
shNC
B

Supplement: Supplementary file 6 — Additional file 6: Transfection effect of Aak1 interference plasmid. A: Transfection effect of shNC observed under a fluorescence microscope (10 × 10). B: Inhibitory effect of the shRNA-positive control (η² = 0.981). C: Effects of shRNA transfection on Aak1 mRNA levels (η² = 0.989). D: Effects of shRNA transfection on Aak1 protein levels (η² = 0.944). Mean ± SD, n = 3, *P < 0.05, **P < 0.01, ***P < 0.001, ****P < 0.0001, ns: not significant. [file 13567_2025_1509_MOESM6_ESM.pptx]

## Slide 1
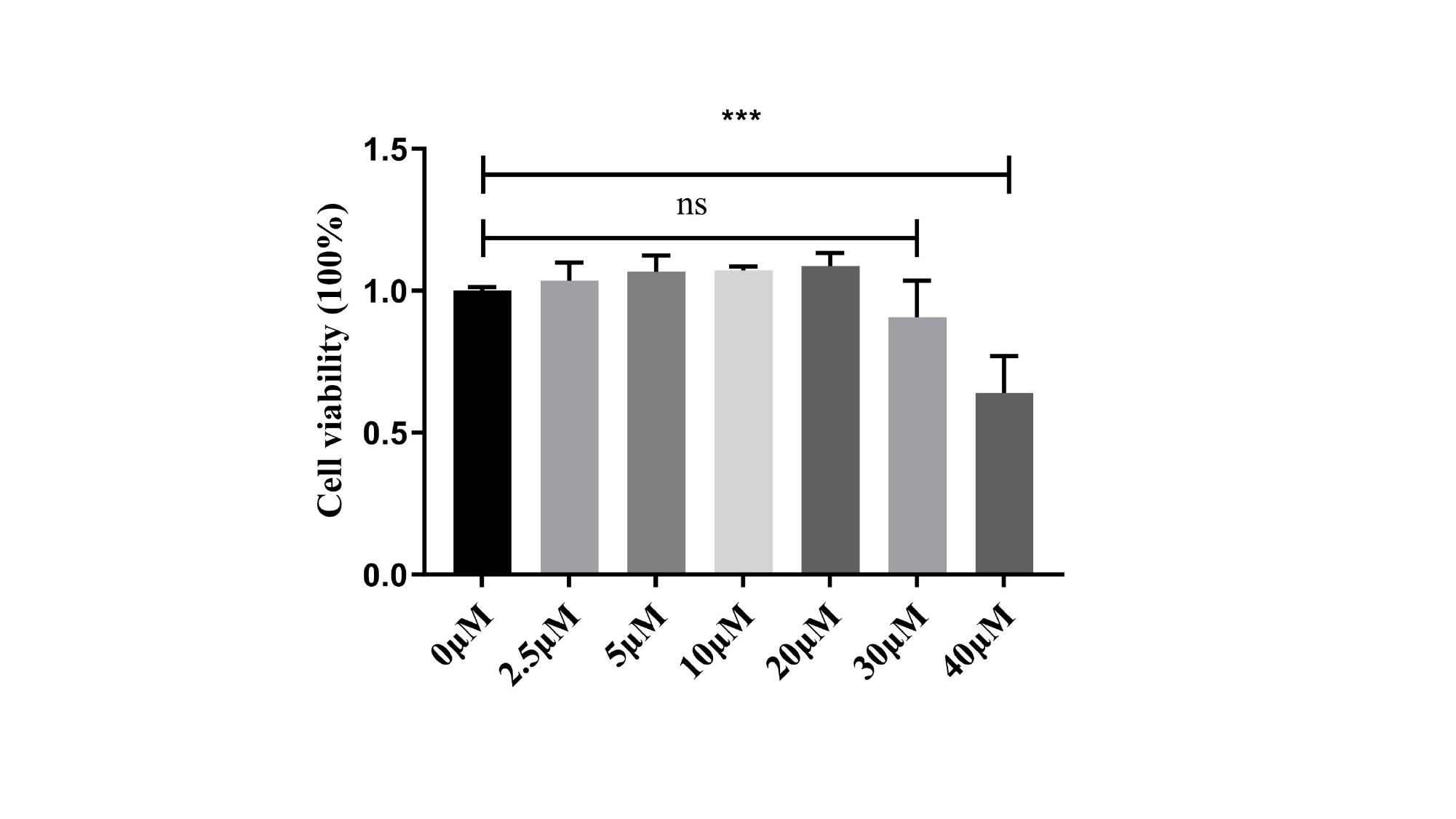

Supplement: Supplementary file 7 — Additional file 7: Toxicity of different concentrations of Cur on PC-12 cells (η² = 0.805). Mean ± SD, n = 3, *P < 0.05, **P < 0.01, ***P < 0.001, ****P < 0.0001, ns: not significant. [file 13567_2025_1509_MOESM7_ESM.pptx]

## Slide 1
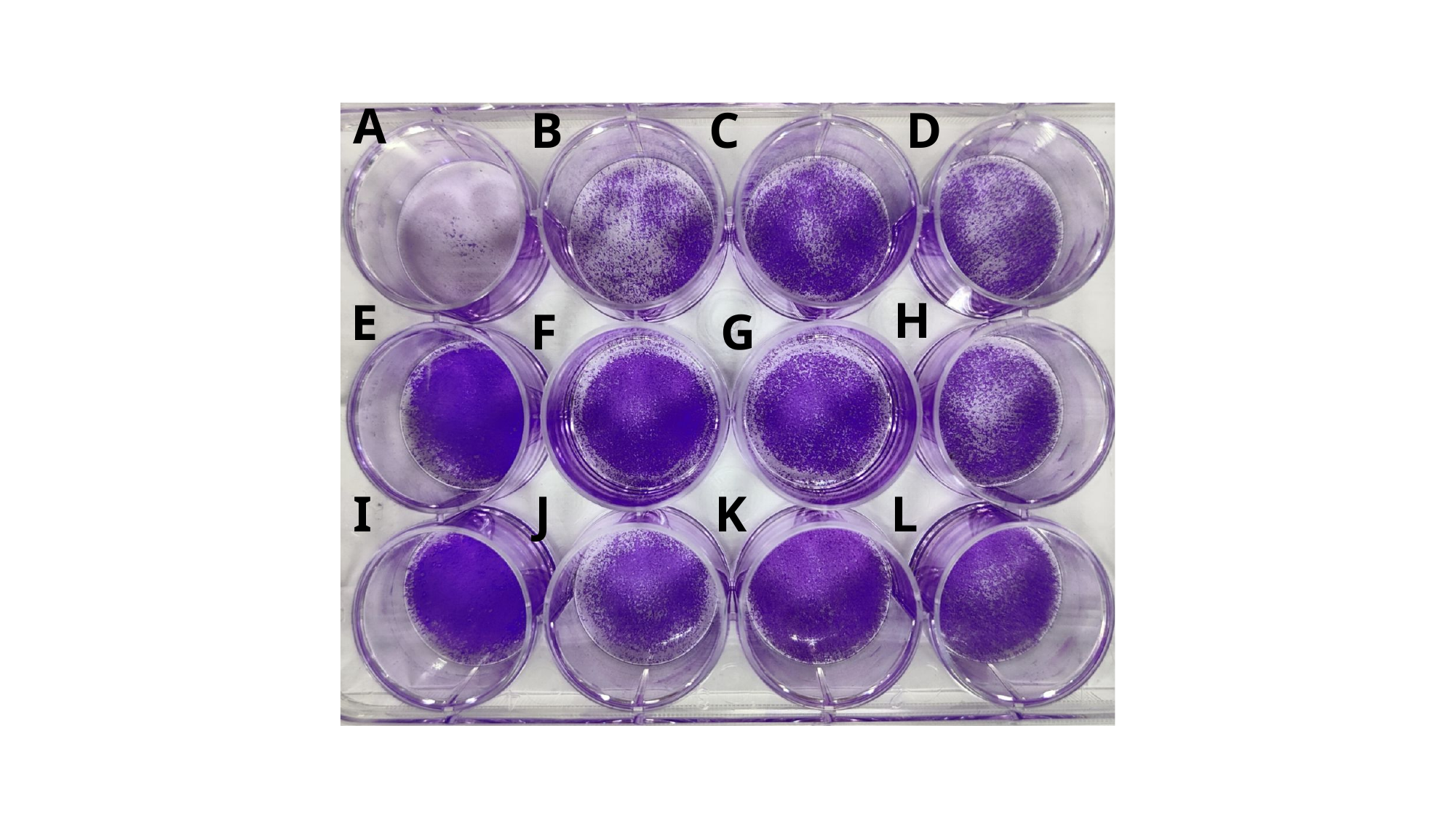

A
B
C
D
H
E
F
G
I
J
K
L

Supplement: Supplementary file 8 — Additional file 8: The concentration of Cur inhibited the virion content in the supernatant of DEX-activated PRV cells. A: PRV with an MOI of 100, directly infected with PK-15. B: PK-15 was infected with the supernatant of PC-12 cells after 24 h of PRV infection with MOI=1 and incubation with 0.5 µM DEX for 4 h. C: PC-12 cells without virus infection were incubated with 0.5 µM DEX for 4 h, and PK-15 was incubated with the culture supernatant. D: PRV with MOI=1 directly infects PK-15. E: PK-15 cells were incubated directly with 5 µM Cur. F: PRV with MOI=1 infected PC-12 cells for 24 h+5 µM Cur incubated PC-12 cells for 24 h+0.5 µM DEX incubated PC-12 cells for 4 h and cultured supernatant incubated PK-15 cells. G: PK-15 cells were incubated directly with 0.5 µM DEX. H: PK-15 cells were infected with PC-12 cell supernatant 24 h after direct incubation of 0.5 µM DEX with PK-MOI=1 PRV. I: PK-15 cells were incubated directly with 10 µM Cur. J: PRV with MOI=1 infected PC-12 cells for 24 h+10 µM Cur incubated PC-12 cells for 24 h+0.5 µM DEX incubated PC-12 cells for 4 h and cultured supernatant incubated PK-15 cells. K: PK-15 cells were incubated with the supernatants of PC-12 cells without viral infection. L: Blank control. [file 13567_2025_1509_MOESM8_ESM.pptx]
